# Anterior Cingulate Cortex Mediates Hyperalgesia and Anxiety Induced by Chronic Pancreatitis in Rats

**DOI:** 10.1007/s12264-021-00800-x

**Published:** 2021-12-15

**Authors:** Dan Ren, Jia-Ni Li, Xin-Tong Qiu, Fa-Ping Wan, Zhen-Yu Wu, Bo-Yuan Fan, Ming-Ming Zhang, Tao Chen, Hui Li, Yang Bai, Yun-Qing Li

**Affiliations:** 1 Department of Anatomy, Guangxi Medical University, Nanning, 510000 China; 2 Department of Anatomy, Histology and Embryology and K. K. Leung Brain Research Centre, Fourth Military Medical University, Xi’an, 710032 China; 3 Department of Anatomy, Xuzhou Medical University, Xuzhou, 221004 China; 4 Department of Cardiology, The Second Affiliated Hospital of Xian Jiaotong University, Xi’an, 710004 China; 5 Department of Neurosurgery, General Hospital of Northern Theater Command, Shenyang, 110016 China; 6 Key Laboratory of Brain Science Research and Transformation in Tropical Environment of Hainan Province, Haikou, 570216 China; 7 Department of Human Anatomy, College of Basic Medicine, Dali University, Dali, 671000 China

**Keywords:** Chronic pancreatitis, Anterior cingulate cortex, Nucleus tractus solitaries, Hyperalgesia, Anxiety

## Abstract

Central sensitization is essential in maintaining chronic pain induced by chronic pancreatitis (CP), but cortical modulation of painful CP remains elusive. Here, we examined the role of the anterior cingulate cortex (ACC) in the pathogenesis of abdominal hyperalgesia in a rat model of CP induced by intraductal administration of trinitrobenzene sulfonic acid (TNBS). TNBS treatment resulted in long-term abdominal hyperalgesia and anxiety in rats. Morphological data indicated that painful CP induced a significant increase in FOS-expressing neurons in the nucleus tractus solitarii (NTS) and ACC, and some FOS-expressing neurons in the NTS projected to the ACC. In addition, a larger portion of ascending fibers from the NTS innervated pyramidal neurons, the neural subpopulation primarily expressing FOS under the condition of painful CP, rather than GABAergic neurons within the ACC. CP rats showed increased expression of vesicular glutamate transporter 1, and increased membrane trafficking and phosphorylation of the *N*-methyl-D-aspartate receptor (NMDAR) subunit NR2B and the α-amino-3-hydroxy-5-methyl-4-isoxazole propionic acid receptor (AMPAR) subunit GluR1 within the ACC. Microinjection of NMDAR and AMPAR antagonists into the ACC to block excitatory synaptic transmission significantly attenuated abdominal hyperalgesia in CP rats, which was similar to the analgesic effect of endomorphins injected into the ACC. Specifically inhibiting the excitability of ACC pyramidal cells *via* chemogenetics reduced both hyperalgesia and comorbid anxiety, whereas activating these neurons *via* optogenetics failed to aggravate hyperalgesia and anxiety in CP rats. Taken together, these findings provide neurocircuit, biochemical, and behavioral evidence for involvement of the ACC in hyperalgesia and anxiety in CP rats, as well as novel insights into the cortical modulation of painful CP, and highlights the ACC as a potential target for neuromodulatory interventions in the treatment of painful CP.

**Supplementary Information:**

The online version contains supplementary material available at 10.1007/s12264-021-00800-x.

## Introduction

Recurrent or constant abdominal pain is the most prominent feature of chronic pancreatitis (CP), and this adversely impacts the quality of life in patients with CP. The pathogenesis of painful CP is poorly understood, and its management has become one of the most challenging issues for both gastroenterologists and pain physicians [1]. It was once believed that pancreatic causes could explain painful CP in most patients; however, some patients still suffer from pain after surgical removal of the pancreatic causes of pain [2, 3]. Thus, the focus of recent research on painful CP has shifted from the anatomical mechanism of the pancreas to the neurobiological mechanisms, which can be summarized in the following three processes: peripheral sensitization, pancreatic neuropathy, and neuroplasticity changes in the central pain circuit [4].

The sensory afferent fibers of the pancreas can be divided into two parts [5]. On one hand, visceral information is transmitted through the greater and lesser splanchnic nerves to the bilateral T4 to L4 segments of the spinal cord. Sensory information enters the spinal dorsal horn and reaches the central nervous system through the spinal lemniscus [6]. This pathway has been widely studied [2, 7]. On the other hand, visceral information is transmitted to the nucleus tractus solitarii (NTS) through the bilateral vagus nerves [8, 9], then is relayed to higher brain centers, such as the locus coeruleus (LC), parabrachial nuclei, rostral ventrolateral medulla, amygdala, and thalamus [6, 10], and ultimately reaches the limbic system and cognitive brain centers, which are thought to mediate the emotional aspects of pain [8].

The cerebral cortex is the ultimate hub for pain perception and sensitization, mediating the discriminative, affective, and cognitive dimensions of pain [11]. Electroencephalogram [12–14] and imaging studies [15, 16] have shown that the insular cortex and anterior cingulate cortex (ACC) play an important role in pain processing under the condition of CP. The ACC is a critical hub for gut-related viscerosensory processing [17] and visceromotor control [18]. In addition, the ACC participates in pain perception and the modulation of emotional pain. Long-term potentiation (LTP) of excitatory synaptic transmission within the ACC is believed to maintain pathological pain and pain-related anxiety [19].

Studies concerning the role of the ACC in chronic visceral pain have been conducted in a rat model of visceral hypersensitivity (VH) [20, 21]. Glutamate is the major excitatory neurotransmitter within the ACC, and it mediates excitatory synaptic transmission *via* the N-methyl-D-aspartate receptor (NMDAR) and the α-amino-3-hydroxy-5-methyl-4-isoxazole propionic acid receptor (AMPAR) [19]. Electrophysiological studies of VH rats have shown that colorectal anaphylaxis leads to enhanced synaptic transmission within the ACC [20, 22, 23] by postsynaptic recruitment of AMPARs [20, 24] and NMDARs [20, 22, 24, 25]. Thus, bilateral ACC lesioning or inactivation attenuates visceral hyperalgesia as well as pain-related aversion in VH rats [20, 21, 26]. Nevertheless, whether such neuroplastic changes occur within the ACC under the condition of painful CP is still unknown. In addition, the neurocircuitry in the ACC that is the basis of the modulation of painful CP has received little attention. Our previous studies support the hypothesis that the NTS is an important central site for the processing of visceral pain caused by CP and angina pectoris [27, 28]. We propose that the ACC may receive direct visceral pain inputs from the NTS, and neuroplastic changes within the ACC are involved in hyperalgesia and comorbid anxiety under the condition of painful CP.

To test our hypothesis, a CP rat model was established by intraductal administration of trinitrobenzene sulfonic acid (TNBS). The abdominal withdrawal threshold (AWT) test was used to measure abdominal hyperalgesia and the open field test to assess anxiety in CP rats. The existence of a direct NTS–ACC pathway and its involvement in painful CP was examined *via* the combination of tract tracing and immunostaining methods, with FOS expression as a bioactive marker for neural activation by painful CP. Morphological and molecular techniques were then applied to study the molecular basis of cortical sensitization in CP rats. Finally, the regulatory role of the ACC in abdominal hyperalgesia and anxiety was assessed *via *a Pharmacological approach, as well as optogenetics and chemogenetics aiming at manipulating the activity of ACC pyramidal neurons in CP rats.

## Materials and Methods

### Animals

Male Sprague-Dawley rats (250–280 g) were provided by the Experimental Animal Center of the Fourth Military Medical University (Xi’an, China). All protocols were approved by the Institutional Animal Care and Use Committee of the Fourth Military Medical University. The rats were habituated to the experimental environment for 3 days before behavioral tests, with feeding in an environment maintained under a 12/12 h light/dark cycle. All behavioral tests were carried out during the light phase.

### Chronic Pancreatitis Rat Model

In accordance with previous reports, CP was induced by infusion of 2% TNBS (Sigma, St. Louis, MO, USA) *via* pancreatic catheter infusion [29]. Rats in the sham group were infused with the same amount of saline. The naïve rats underwent no surgery.

### Behavioral Tests

Behavioral tests were performed on postoperative days (POD) 3, 7, 14, and 28. The abdominal withdrawal threshold (AWT), a measure of referred abdominal mechanical hypersensitivity, is an indirect marker of visceral sensitization [29]. The AWT was calculated using von Frey filaments (VFFs; Stoelting, Kiel, WI, USA). The abdominal area designated for stimulation was shaved before tests. The rats were handled with caution and habituated to the testing apparatus for at least 30 min until they calmed down for 3 consecutive days before testing. VFFs with increasing force (0.16–26 g) were applied vertically to the right upper abdomen 5 times, each for 5–8s at 5-min intervals, and the minimal force that elicited a withdrawal response at least 3 times was considered as the AWT. The hindpaw withdrawal threshold (PWT) was also measured as in our previous work [30].

Behavioral tests for higher brain function were conducted as described in previous studies [31, 32]. In the open field test, rats were acclimated to the observation room, and then placed in the center of a novel open field (100 cm × 100 cm × 60 cm). A motion-tracking system (Shanghai Mobile Datum Information Technology, Co. Ltd, Shanghai, China) was used to record their locomotion for 15 min. Anxiety-like behavior was estimated by the total distance traveled (total distance) and the percentage of that distance spent in the center of the open field (% center distance).

In the elevated plus maze test (Med Associates, St. Albans, Vermont, USA), rats were placed in the center square (10 cm × 10 cm) with the head toward a closed arm (50 cm × 10 cm) and recorded for 5 min with the motion-tracking system. Anxiety-like behavior was evaluated by the number of arm entries (total crossings) and the time spent in the open arms (% time in open arms).

The rotarod test (Shanghai Biowill Co. Ltd, Shanghai, China) was used to assess motor coordination. Before tests, rats were trained in three trials with the rod rotating at a constant speed of 5 revolutions per minute (r/min). During tests, rats were placed on the rotarod apparatus, with the rotation speed starting at 3 r/min and progressing to a maximum of 30 r/min. The latency to falling was recorded to assess motor coordination.

The forced swimming test was conducted to evaluate depression-like behavior. Rats were forced to swim in an open plastic cylinder (diameter 28 cm, height 50 cm) filled with water (maintained at 25 °C) to a height of 35 cm. The duration of immobility (floating without any struggle or active movements with the forepaws) was measured during a 5-min session.

### Hematoxylin and Eosin Staining of Pancreatic Tissue

After behavioral tests, the rats were deeply anesthetized with 2% sodium pentobarbital (60 mg/kg) injected into the abdominal cavity, and pancreatic tissue obtained to confirm pancreatitis. The pancreatic tissue was fixed in 4% paraformaldehyde in phosphate-buffered saline (PBS, pH 7.2–7.4) overnight at 4 °C, transferred to progressive xylene washes, and embedded in paraffin. The paraffin blocks were cut into 5 μm sections and stained with hematoxylin and eosin (H&E).

### Brain Stereotaxic Injection

Each rat was anesthetized with 2% sodium pentobarbital (40 mg/kg) injected into the abdominal cavity and then fixed on a stereotaxic apparatus (68025, RWD Life Science, Shenzhen, China). After routine disinfection, a hole was drilled the skull with an electric cranial drill. For anterograde labeling, 0.2 μL rAAV-CaMKIIα-EYFP-WPRE-pA (rAAV9-107-1-3, BrainVTA, Wuhan, China) was injected into the left NTS with at the following coordinates: anterior-posterior, AP: –14.16; medial-lateral, ML: ±0.30; dorsal-ventral, DV: +7.80 mm. For retrograde labeling, 0.02 μL of 4% fluorogold (FG; 80014, Fluorochrome, Denver, CO, USA) dissolved in 0.9% saline was injected into the right ACC (AP: + 1.20; ML: ± 0.60; DV: − 2.80 mm). The injections were performed using a microinjection pump for ~ 15 min. Injection needles were removed 5 min after injection. The scalp was then sutured. Each rat was returned to its home cage and fed for 4 weeks (for virus injection) or 1 week (for FG injection) before perfusion. The injection sites were observed under a FluoView1000 confocal microscope (Olympus, Shinjuku City, Tokyo, Japan).

### Immunostaining

#### Single Immunostaining of VGluT1 or FOS

TNBS- and saline-treated rats were deeply anesthetized as described above and perfused for FOS or vesicular glutamate transporter 1 (VGluT1) immunohistochemistry. After perfusion, the brains were removed, placed in 30% sucrose solution at 4 °C until they sank, and then cut into 40 µm thick coronal sections. Sections containing the ACC and NTS were incubated in 10% normal donkey serum (NDS) for 40 min at 26 °C to block non-specific immunoreactivity.

To confirm activation of the ACC and NTS by painful CP, single FOS immunostaining in tissue from sham rats (POD 14) and CP rats at different time points (POD 3, 7, 14, and 28) was performed according to a previously-described protocol [31]. After blocking, the sections were incubated overnight at 4 °C for 18–24 h with mouse anti-FOS (1:500; ab11959, Abcam, Cambridge, MA, USA) and biotinylated donkey anti-mouse secondary antibody (1:500; AP192B, Millipore, Billerica, MA, USA) for 6 h (for immunofluorescent staining) at room temperature, and avidin-biotin complex (1:200; PK-6101, Vector labs, Burlingame, CA, USA) for 2 h at 26 °C. The sections were then immersed in 0.05 mol/L Tris-HCl (pH 7.6) containing 0.02% diaminobenzidine tetrahydrochloride (D006, Dojin Laboratory, Kumamoto, Japan) and 0.003% H_2_O_2_ for the visualization of FOS staining. The average number of FOS-expressing neurons in three sections containing the ACC or NTS from each rat in the sham and CP groups was counted under a 20× objective lens (*n* = 3 rats per group).

To substantiate enhanced glutamatergic transmission within the ACC, double VGluT1/NeuN immunostaining in tissue from sham and CP rats on POD 14 was applied as follows: the sections were removed from the NDS and incubated in mouse anti-NeuN (1:300; MAB324-K, Millipore) and rabbit anti-VGluT1 (1:500; 135 303, Synaptic Systems, Göttingen, NI, Germany) overnight at 4 °C. The sections were then incubated in Alexa594-donkey anti-mouse (1:500; A21203, Invitrogen, Carlsbad, CA, USA) and Alexa488-donkey anti-rabbit (1:500; A21206, Invitrogen) for 4 h at 4 °C in darkness.

After each step, the sections were rinsed three times with 0.01 mol/L PBS for 10 min. After the final step, the sections were mounted on microscope slides and cover-slipped for examination by light microscopy (AH-3, Olympus, Tokyo, Japan) for FOS staining or by confocal microscopy (CLSM, FV1000, Olympus) for VGluT1/NeuN double-labeling immunofluorescent staining. Images were analyzed using ImageJ2 or Fluoview software (Olympus).

#### Double Immunostaining of CaMKII (or GAD67) and FOS

To explore whether ACC glutamatergic and GABAergic neurons are innervated by a direct NTS–ACC pathway, single immunostaining of CaMKII or GAD67 was first performed in the ACC in CP rats four weeks after rAAV-CaMKIIα-EYFP injection into the NTS. Two weeks after virus injection, the rats were treated with TNBS to establish the CP model. Brain sections were prepared as described previously. After confirmation of the injection site within the NTS, virus-labeled terminals fibers within the ACC were observed, and then representative images indicating the injection site and projection area were captured under the confocal microscope. Next, the targeted area in ACC was immunostained with the antibodies and procedures as follows: after blocking, the ACC sections were incubated overnight at 4 °C in mouse anti-GAD67 (1:300; ab26116, Abcam) or rabbit anti-CaMKII (1:300; ab134041, Abcam). The sections were then incubated with Alexa594-donkey anti-mouse (1:500; A21203, Invitrogen) or Alexa594-donkey anti-rabbit (1:500; A21207, Invitrogen) for 4 h at 4 °C in darkness. The close contacts between EYFP-labeled terminals and CaMKII or GAD67-expressing cell bodies within the ACC were observed under the confocal microscope.

To explore whether ACC glutamatergic and GABAergic neurons are activated by painful CP, double immunostaining of CaMKII and FOS, as well as GAD67 and FOS were further performed within the ACC in the naïve, sham, and CP groups using the antibodies and procedures as follows: after blocking, the ACC sections were incubated in rabbit anti-CaMKII (1:300; ab134041, Abcam) and mouse anti-FOS (1:500; ab11959, Abcam) for double CaMKII/FOS immunostaining, and in rabbit anti-GAD67 (1:300; ab97739, Abcam) and mouse anti-FOS (1:500; ab11959, Abcam) for double GAD67/FOS immunostaining overnight at 4 °C. The sections were then incubated with Alexa594-donkey anti-mouse (1:500; A21203, Invitrogen) and Alexa488-donkey anti-rabbit (1:500; A21206, Invitrogen) (for double CaMKII/FOS immunostaining), or Alexa594-donkey anti-mouse (1:500; A21203, Invitrogen) and Alexa488-donkey anti-rabbit (1:500; A21206, Invitrogen) (for double GAD67/FOS immunostaining) for 4 h at 4 °C in darkness. The expression of FOS in CaMKII or GAD67-ir neurons in the ACC was observed under the confocal microscope. Three sections of the ACC from each rat were counted under a 20× objective lens to obtain the average number of FOS-expressing neurons among the different groups (*n* = 4 rats per group). For double-labeled neurons, the total number of double-labeled neurons and CaMKII or GAD67-expressing neurons from three sections in each rat was counted under a 20× objective lens) and then the proportions of double-labeled neurons to CaMKII or GAD67-expressing neurons were calculated. Furthermore, the average proportion of double-labeled neurons to CaMKII or GAD67-expressing neurons in all rats (*n* = 4) was calculated and compared.

#### Double Immunostaining of FG and FOS

To explore whether neurons in the NTS projecting to the ACC were activated by painful CP, double immunostaining of FG and FOS was performed in the NTS of CP rats 1 week after FG injection into the ACC. TNBS was administered 1 week before FG injection to establish the CP model. The preparation of brain sections was as previously described. After confirmation of the injection site in the ACC and virus-labeled cell bodies in the NTS, immunostaining was performed using the following antibodies and procedures: after blocking, the ACC sections were incubated in mouse anti-FOS (1:500; ab11959, Abcam) and guinea pig anti-FG (1:200, NM-101, ProtosBiotech, New York, NY, USA) overnight at 4 °C. The sections were then incubated with Alexa594-donkey anti-mouse (1:500; A21203, Invitrogen) or Alexa488-donkey anti-guinea pig (1:500; 706-545-148, Jackson Immunoresearch, West Grove, PA, USA) for 4 h at 4 °C in darkness. The expression of FOS in FG-labeled neurons in the NTS was observed under the confocal microscope.

### Western Blot Analysis

The ACC of each rat was immediately removed into cold artificial cerebrospinal fluid and homogenized in lysis buffer containing proteinase inhibitors (REF04693159001, Roche, Basel, Switzerland) and phosphatase inhibitors (REF04906837001, Roche). Total protein (for VGluT1 assay) was prepared according to the steps described in our previous study [30]. Membrane and cytoplasmic proteins were isolated using the procedure of the Minute™ Plasma Membrane Protein Isolation Kit (SM-005, Invent Biotechnologies, Eden Prairie, MN, USA). Protein concentration was quantified using a bicinchoninic acid kit (Thermo Scientific™, Rockford, IL, USA). The antibodies used and their concentrations were as follows: mouse anti-VGluT1 (1:500; MAB5502, Millipore), rabbit anti-pNR2B-Tyr^1472^ (1:500; AB5403, Millipore), rabbit anti-NR2B (1:500; ab65783, Abcam), rabbit anti-GluR1 (1:500; AB2285, Millipore), rabbit anti-pGluR1-Ser^845^ (1:500; PA5110124, Invitrogen), rabbit anti-β-actin (1:5000; A1978, Sigma), and rabbit anti-N-cadherin (1:2000; AB1550, Millipore), and horseradish peroxidase-conjugated secondary antibodies (goat anti-rabbit, 1:5000; ZB-2301, Zsgb Biotech, Los Altos, CA; and goat anti-mouse, 1:5000; ZB-2305, Zsgb Biotech). The protein bands were visualized using an enhanced chemiluminescence kit (Pierce, Rockford, IL, USA) and scanned using the ChemiDoc Imaging System (Bio-Rad Richmond, CA, USA). The band intensities were quantified using ImageJ, with all samples normalized using β-actin or N-cadherin as loading controls.

### Cannulation Surgery and Drug Microinjection

One week before surgery, both TNBS and sham rats were anesthetized and secured on a stereotaxic frame (RWD, Shenzhen, China). A double guide cannula (26 gauge) was implanted bilaterally into the ACC (AP: +1.20, ML: ±0.60, DV: –2.50 mm). Before establishing the CP model, baseline AWT tests were run. On POD 13, AWT tests were run to verify the successful establishment of the painful CP model. On POD 14, intra-ACC injections were delivered through a double injector cannula (30 gauge), located 0.2 mm below the guide. A Hamilton syringe (10 μL) was connected to the injector by a thin polyethylene tube and driven by a motorized pump (Alcbio, Shanghai, China). Amino-5-phosphonovaleric acid (AP-5, 50 mmol/L, 0.4μL, per side; Tocris, Bristol, UK) or cyano-2, 6-cyano-7-nitroquinoxaline-2,3-dione (CNQX, 20 mol/L, 0.4μL, per side; Tocris) was infused into the bilateral ACC at 0.05 μL/min with the same volume of saline in control rats. The injection cannula was removed 5 min after completing the injection. AWT was measured 30 min after microinjection. In addition, the analgesic effects of opioid injection into the ACC were tested as a positive control to evaluate the analgesic effects of CNQX and AP-5 in CP rats. Morphine (25 μg/0.4 μL per side; PH014355, Sigma), endomorphin-1 (EM-1; 25 μg/0.4 μL per side; 1055, Tocris), endomorphin-2 (EM-2; 25 μg/0.4 μL per side; E3148, Sigma), or vehicle (saline; 0.4 μL per side;) were microinjected bilaterally into the ACC 30 min before behavioral tests on POD 3, 5, 7, 10, and 14. The injection sites were verified *post hoc*, and rats with inaccurate sites were eliminated from the study.

### Optogenetics, Chemogenetics, and Behavioral Tests

In optogenetic tests, rAAV-CaMKIIα-ChR2-mCherry (0.4 μL/30 min) was injected into the bilateral ACC (AP: + 1.20, ML: ± 0.60, DV: − 2.80 mm) one week before CP induction. One week after injection, baseline AWT was measured, and then a single optic fiber was implanted over the virus injection site (AP: + 1.20, ML: 0.00, DV: − 2.50 mm) and then fixed with dental cement. On POD 14, blue light (5 mW, 10-ms pulses at 10 Hz, 473 nm) was delivered for 15 min (5 min on, 5 min off, and 5 min on) using a laser source (Aurora-220, Newdoon Technology, Hangzhou, China). AWT was assessed before, during, and after illumination. Following the AWT tests, the open field test was run for 15 min, and blue light was delivered from the fifth to the tenth minute.

In chemogenetic tests, rAAV-CaMKIIa-hM4Di-mCherry (0.4 μL/30 min) was injected into the bilateral ACC one week before baseline AWT testing and CP induction. On POD 13, AWT tests were run to verify successful establishment of the painful CP model. On POD 14, either saline or clozapine-N-oxide (CNO, 3 mg/kg; C4759, LKT Laboratories, MN, USA) was administered intraperitoneally, and AWT tests were performed 1 h later. Following AWT tests, the open field test was run for 15 min. The injection sites were verified *post hoc* for both optogenetic and chemogenetic tests, and rats with inaccurate injection sites were eliminated from the study.

### Data Analysis

Data were analyzed using the SPSS ver. 19.0 (IBM Corp., Armonk, NY, USA) and are expressed as the mean ± standard error (mean ± SEM). Images were processed using Adobe Photoshop CS5 (Mountain View, CA, USA). Statistical comparisons between multiple groups were made using one-way ANOVA or one-way repeated ANOVA followed by the LSD *post-hoc* test, while statistical comparisons between two groups were made using unpaired or paired *t*-tests. Statistical graphics were produced using GraphPad Prism Version 5 (San Diego, CA, USA). *P* < 0.05 was considered statistically significant.

## Results

### TNBS-Induced Abdominal Hyperalgesia and Hypolocomotion

**Fig. 1:**
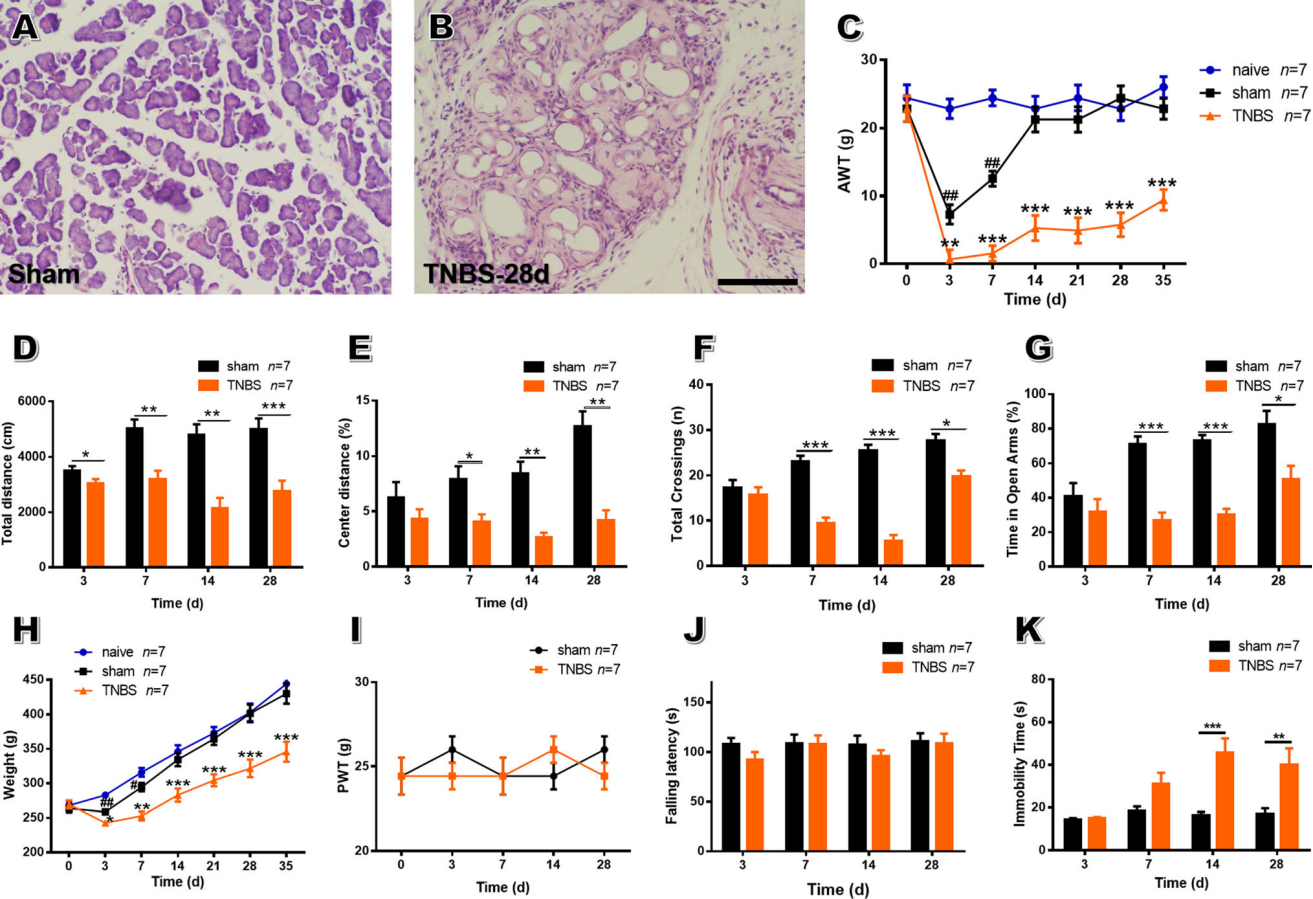
Chronic pancreatitis induced by TNBS elicits abdominal hyperalgesia and anxiety-like behaviors in rats. **A**, **B** Representative H&E-stained histological sections of the pancreas on POD 28 in sham and TNBS-treated rats (scale bar, 100 μm). **C** TNBS-treated rats show a decreased abdomen withdrawal threshold during the course of CP compared to sham rats, while sham rats exhibit transient abdomen mechanical hypersensitivity that returns to baseline on POD 14 (*n* = 7 rats per group; **P < 0.01, ***P < 0.001, TNBS *vs* sham; ^##^*P* < 0.01, sham *vs* naïve, one-way repeated ANOVA). **D**, **E** CP rats travel shorter distances (**D**) from POD 3 to 28 and travel less distance in the center area (**E**) from POD 7 to 28 in the open field test than sham rats. **F**, **G** CP rats exhibit fewer crossings (**F**) and less time in the open arms (**G**) in the high elevated maze test than sham rats from POD 3 to 28 (*n* = 7 per group; *P < 0.05, **P < 0.01, ***P < 0.001, TNBS *vs* sham, unpaired *t*-test). **H** CP rats lose body weight during the course of CP compared to sham rats, while sham rats show a transient weight loss from POD 3 to 7 which returns to baseline after POD 14 (*n* = 7 per group; *P < 0.05, **P < 0.01, ***P < 0.001, TNBS *vs* sham; ^#^*P* < 0.05, ^##^*P* < 0.01, sham *vs* naïve, one-way repeated ANOVA). **I** CP rats exhibit no significant change in hindpaw withdrawal threshold compared with the sham group (*P* > 0.05, *n* = 7 per group, one-way repeated ANOVA). **J**, **K** No change in falling latency between CP and sham rats in the rotarod test (**J**), while CP rats exhibited a prolonged immobility time in the forced swimming test from POD 14 to 28 (**K**) (*n* = 7 per group; **P < 0.01, ***P < 0.001, TNBS *vs* sham, unpaired *t*-test). AWT, abdomen withdrawal threshold; PWT, paw withdrawal threshold; TNBS, trinitrobenzene sulfonic acid.

The TNBS-induced CP rat model is commonly used in experimental CP studies [33]. The validity of the CP model was evidenced by histopathological changes in pancreatic sections, such as acinar atrophy, inflammatory infiltration, and stromal fibrosis (Fig. 1A, B), together with weight loss (Fig. 1H).

Sham rats exhibited a transient decrease of AWT, which reached a minimum on POD 3 and returned to baseline on POD 14. This phenomenon was also seen in our previous study, and was owing to post-operative pain [34]. Compared to sham rats, CP rats had a lower AWT from POD 7 to 35 (Fig. 1C), indicating the presence of long-term abdominal hypersensitivity induced by TNBS. Open field testing showed that CP rats traveled shorter distance in the open field from POD 3 to 28 than sham rats, indicating hypolocomotion (Fig. 1D). Importantly, CP rats also traveled less distance in the central area, suggesting the occurrence of anxiety-like emotions (Fig. 1E). Elevated plus maze testing further showed that CP rats had fewer total crossings and spent less time in the open arms (Fig. 1F, G), in accordance with the results of the open field tests.

Several scenarios, such as mood disturbance and motor defects, are worth considering with regard to hypolocomotion in the condition of chronic pain [33]. Thus, several behavioral assays were run to dissect the cause of immobility. CP rats displayed no marked alterations in the PWT compared to the sham group, thus excluding the possibility of somatic hyperalgesia (Fig. 1I). Rotarod testing showed no significant differences in the falling latency at each time point between sham and CP rats, excluding the possibility of motor coordination disorder (Fig. 1J). Interestingly, CP rats exhibited a prolonged immobility time in the forced swimming test compared to the sham group, implying the existence of depression (Fig. 1K). Considering these results, we believe that the hypolocomotion in CP rats is closely related to abdominal hyperalgesia-induced negative emotions, rather than motor disorders or hindlimb pain. Thus, we used both distance traveled and exploratory behavior in the open field test to assess the role of the ACC in emotional pain modulation in CP rats.

### Chronic Pancreatitis Stimuli Induce FOS Expression in the NTS, ACC, and NTS–ACC Projection Neurons

**Fig. 2:**
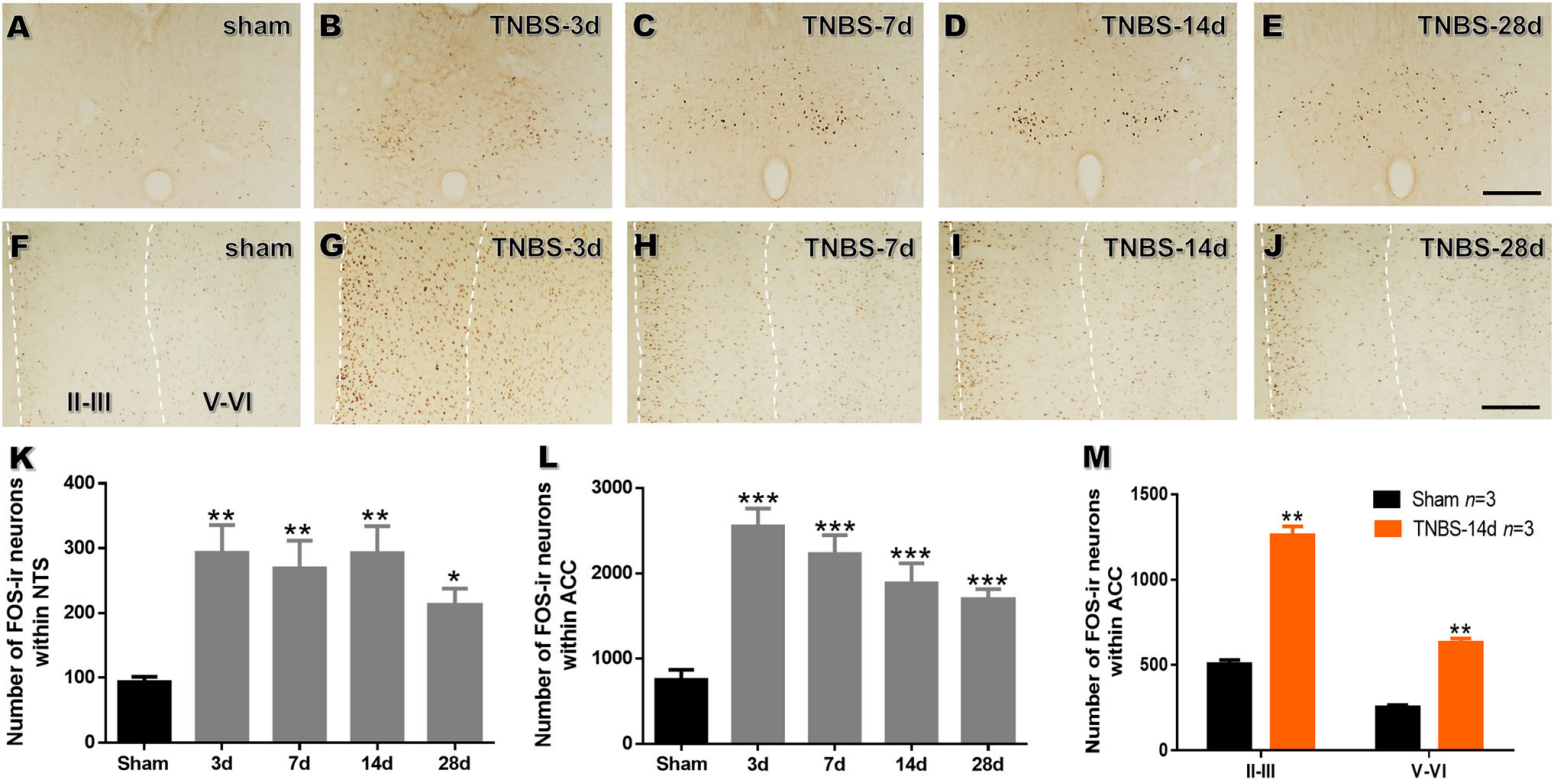
TNBS treatment increases the number of FOS-ir neurons in the NTS and ACC. **A**–**J** Immunochemical staining of FOS in the NTS (**A**–**E**) and ACC (**F**–**J**) in sham rats on POD 14 and in TNBS-treated rats on POD 3, 7, 14, and 28 (scale bars, 200 μm). **K**, **L** Numbers of FOS-ir neurons in the NTS (**K**) and ACC (**L**) in sham rats on POD 14 and in TNBS-treated rats on POD 3, 7, 14, and 28. **M** Numbers of FOS-ir neurons in different layers of the ACC in sham rats and TNBS-treated rats on POD 14 (*n* = 3 per group; *P < 0.05, **P < 0.01, *** *P* < 0.001, TNBS *vs* sham, one-way ANOVA in **K**, **L**, unpaired *t*-test in **M**.

Since FOS expression is strongly correlated with noxious somatic or visceral stimuli [35], we performed FOS immunostaining in the NTS and ACC after the induction of CP. From POD 3 to POD 28, the number of FOS-immunoreactive (-ir) neurons in both the NTS and ACC significantly increased in CP rats compared to that in the sham group (Fig. 2A–L). Further analysis showed that TNBS treatment significantly increased FOS expression in both superficial layers II-III (TNBS: 1260.78 ± 151.48 *versus* sham: 503.89 ± 75.07, *P* < 0.01) and deeper layers V-VI (TNBS: 630.33 ± 75.66 *versus* sham: 252.11 ± 37.54, *P* < 0.01; Fig. 2M). These data laid a morphological foundation for activation of the NTS and ACC by painful CP.

**Fig. 3:**
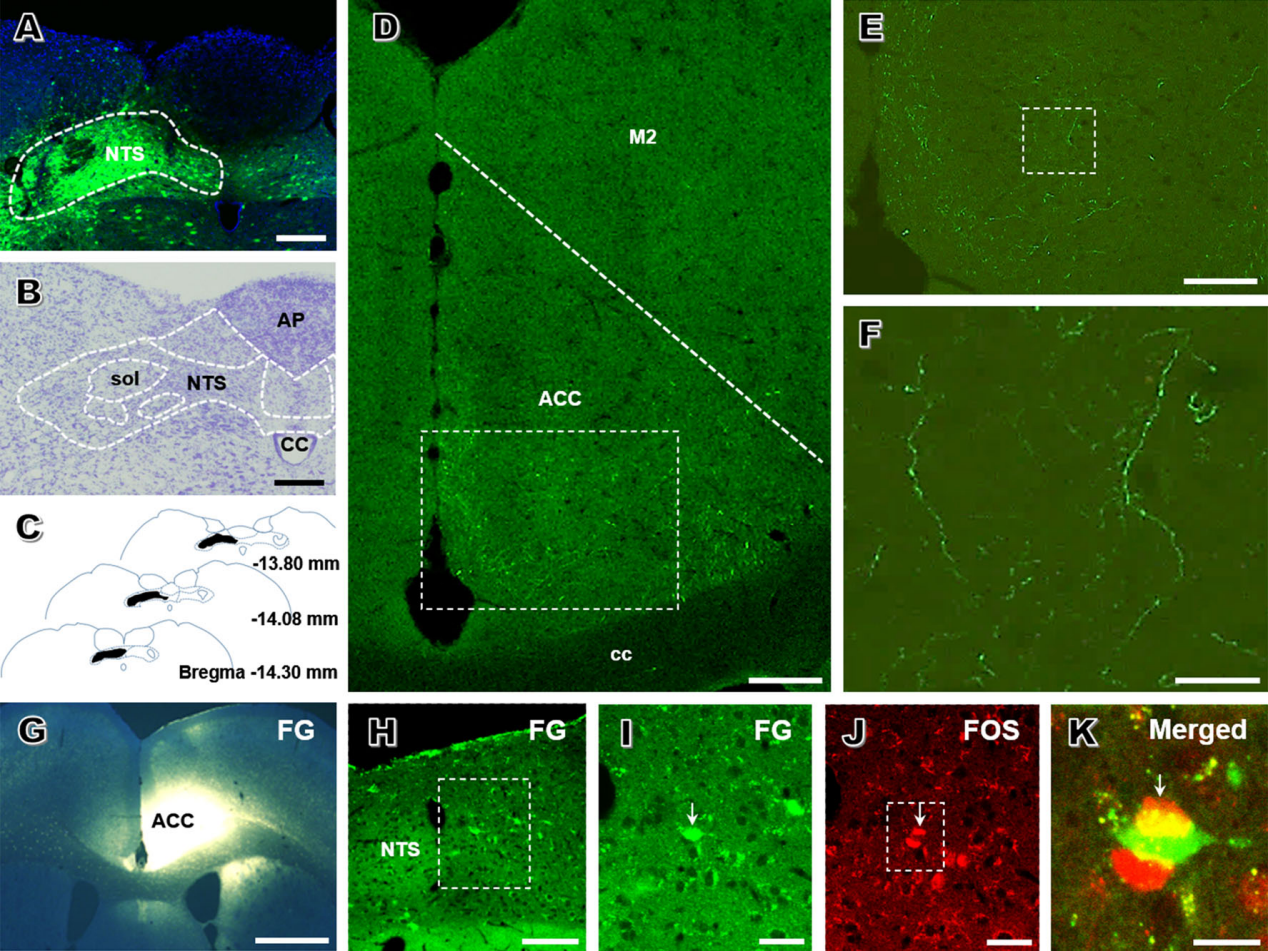
The NTS-ACC pathway is activated in rats with chronic pancreatitis. **A** Photomicrograph of an injection site of rAAV-CaMKIIα-EYFP in the left NTS (scale bar, 200 μm). **B** Representative Nissl-stained section showing an injection site in the NTS (scale bar, 400 μm). **C** Tracings showing the rostrocaudal extent of an injection site at different levels (black areas). **D–F** Representative fluorescence photomicrographs showing anterograde-labeled fibers and terminals in the ACC. The framed area in **D** is magnified in **E**, while the framed area in **E** is enlarged in **F** [scale bars, 350 μm (**D**), 100 μm (**E**), and 40 μm (**F**)]. **G** Photomicrograph of an injection site of FG in the right ACC (scale bar, 400 μm). **H**, **I** Representative fluorescence photomicrographs showing retrograde-labeled ACC-projecting neurons in the NTS [scale bars, 125 μm (**H**) and 40 μm (**I**)]. **I**, **J** Enlargements of the framed area in **H** showing that FG-labeled NTS-ACC projection neurons (**I**, green) express FOS (**J**, red) under painful CP (scale bars, 40 μm). **K** Enlargement of the framed area in (**J**) showing FG and FOS double-labeled neurons (yellow) in the NTS (scale bar, 25 μm). ACC, anterior cingulate cortex; AP, area postrema; cc, corpus callosum; CC, central canal; FG, fluorogold; M2, secondary motor cortex; NTS, nucleus tractus solitarii; sol, solitary tract.

To determine whether the ACC receives direct input from the NTS, the anterograde tracer virus rAAV-CaMKIIα-EYFP was injected into the left NTS. The injection site was mainly located within the NTS with minimal spread to surrounding areas (Fig. 3A–C). EYFP-labeled fibers and terminals were observed in the bilateral ACC, with a dominant distribution on the contralateral side. The fibers and terminals were scattered throughout the superficial and deeper layers of the ACC (Fig. 3D–F).

Double immunostaining results further showed that EYFP-labeled fibers and terminals were more likely to be opposed to CaMKII-expressing neurons than GAD-67-expressing neurons in the ACC (Fig. S1). To determine the expression of FOS in ACC CaMKII and GAD67-expressing neurons under painful CP, double immunostaining of FOS and CaMKII, as well as FOS and GAD67 was performed. We observed that a significantly larger proportion of CaMKII-expressing neurons expressed FOS than GAD67-expressing neurons in the ACC of CP rats (CaMKII: 95.22 ± 1.37% *versus* GAD67: 9.72 ± 1.43%; *n* = 4 per group; *P* < 0.001; Fig. S2). These morphological data suggest that postsynaptic pyramidal neurons in the ACC play a predominant role in the transmission or modulation of painful CP.

Finally, retrograde labeling was used to verify the involvement of the direct NTS–ACC pathway in painful CP. After injection of FG into the ACC in CP rats (Fig. 3G), retrogradely-labeled cell bodies were seen in the contralateral NTS. Interestingly, these neurons expressed FOS (Fig. 3H–K), suggesting that the NTS neurons projecting to the ACC are activated under the condition of painful CP.

### Increased Expression of VGluT1 and Glutamate Receptors in the ACC After TNBS Injection

**Fig. 4:**
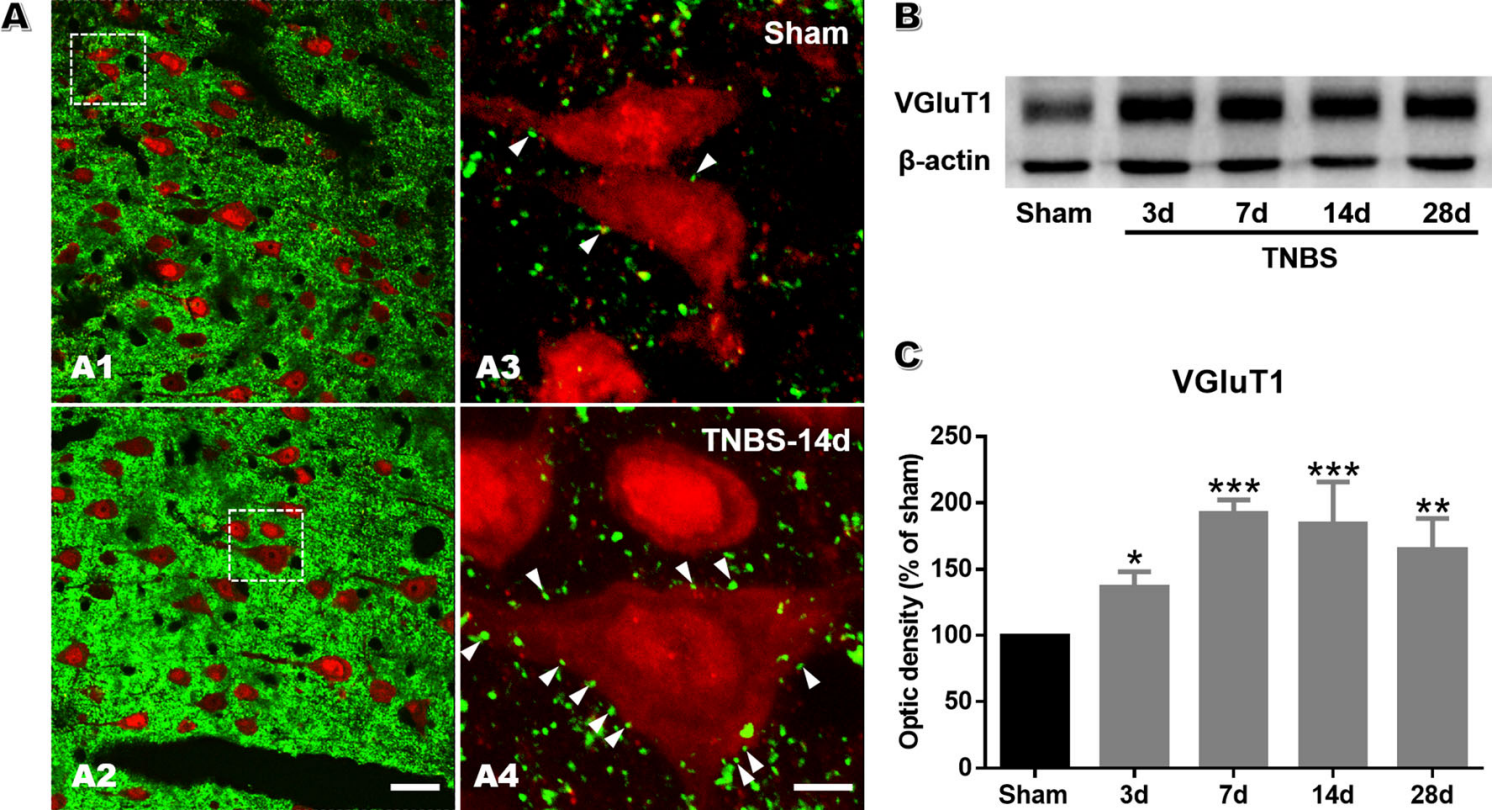
TNBS treatment enhances the expression of VGluT1 in the ACC. **A** Microphotographs showing double-immunofluorescence staining for VGluT1 (green) and NeuN (red) immunoreactivity in the ACC. The framed areas in images **A1** and **A2** are magnified in images **A3** and **A4**. The white arrowheads indicate, VGluT1-labeled axon terminals in close contact with NeuN-labeled somatic or dendritic profiles in the ACC (scale bars, 25 μm in **A1, A2** and 5 μm in **A3, A4**). **B** Representative western blots for VGluT1 in the ACC in sham rats on POD 14 and in TNBS-treated rats on POD 3, 7, 14, and 28. **C** The expression of VGluT1 is significantly enhanced from POD 3 to 28 in TNBS-treated rats compared with sham rats (*n* = 3 per group; *P < 0.05, **P < 0.01, ***P < 0.001, TNBS *vs* sham, one-way ANOVA followed by LSD *post-hoc* test).

VGluTs package glutamate into vesicles for synaptic release and transmission [36]. The expression level of VGluTs determines the amount of glutamate loaded into vesicles and released into the synaptic cleft, thus regulating the efficacy of neurotransmission [36]. Among all the VGluTs, VGluT1 is strongly expressed in the cerebral cortex [37]. In this study, we applied immunostaining and biochemical analyses to examine the expression of VGluT1 within the ACC. Double immunostaining of VGluT1 and NeuN showed that more VGluT1-ir axon terminals appeared to be in close contact with neurons in CP rats than in sham rats on POD 14 (Fig. 4A). The upregulated expression of VGluT1 during the course of CP was then confirmed by western blotting (Fig. 4B–C). These results suggest that CP stimulates glutamate release, providing evidence for enhanced presynaptic transmission in the ACC.

**Fig. 5:**
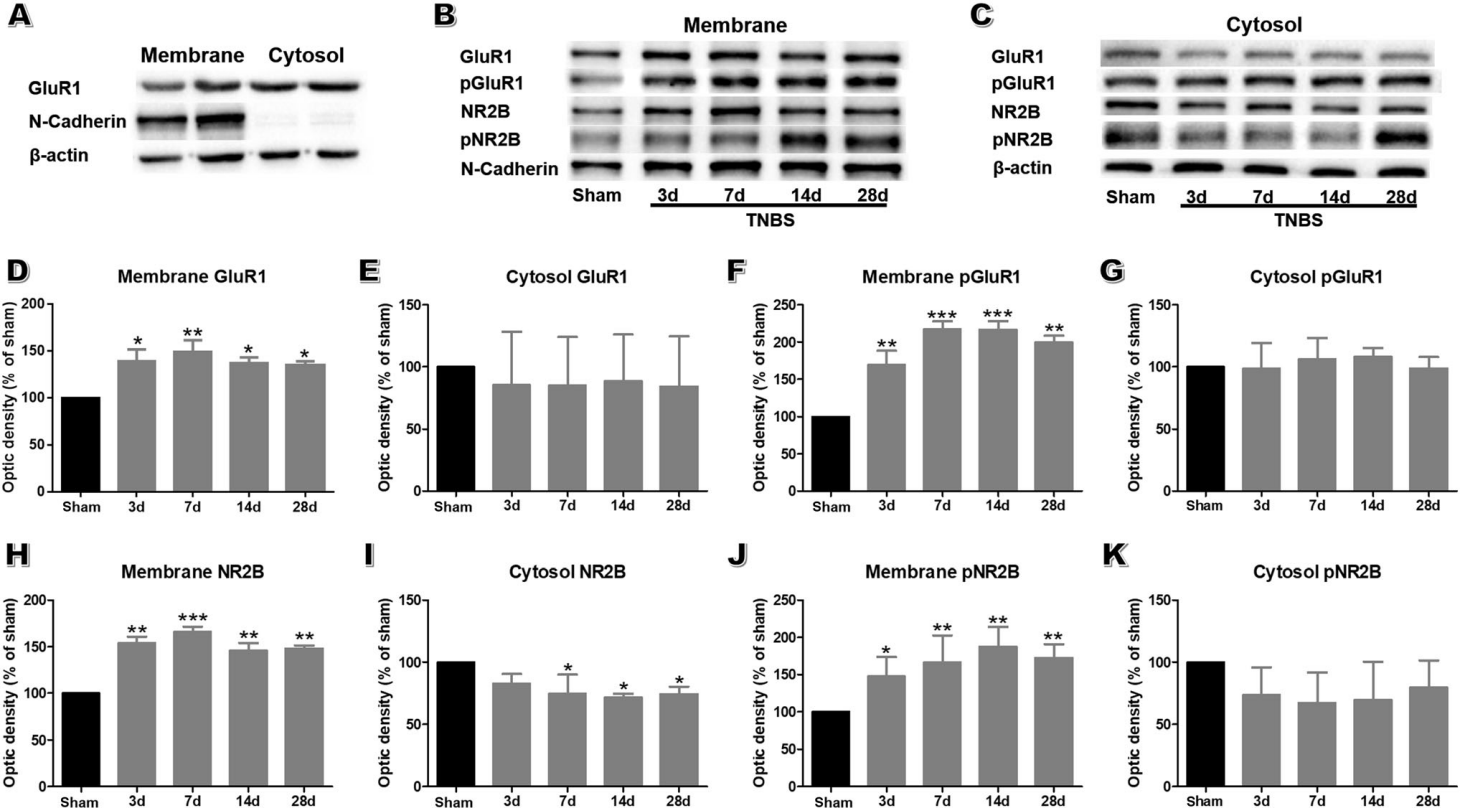
TNBS treatment facilitates the trafficking of glutamate receptor subunits into membrane and their phosphorylation in the ACC. **A** Fractionation of ACC probed for N-cadherin and β-actin to verify the accuracy of the subcellular fractionation procedure. **B, C** Representative western blots for membrane (**B**) and cytosol (**C**) GluR1, pGluR1, NR2B, and pNR2B in the ACC of sham rats on POD 14 and in TNBS-treated rats on POD 3, 7, 14, and 28. **D**, **E** The expression of membrane GluR1 is significantly enhanced after TNBS injection on POD 3, 7, 14, and 28, while cytosol GluR1 does not change. **F**, **G** The expression of membrane pGluR1 is significantly enhanced after TNBS injection on POD 3, 7, 14, and 28, while cytosol pGluR1 does not change. **H**, **I** Membrane NR2B is significantly enhanced after TNBS injection on POD 3, 7, 14, and 28, while cytosol NR2B is significantly decreased on POD 7, 14, and 28. **J**, **K** The expression of membrane pNR2B is significantly increased at all time points in the TNBS-treated group, but that of cytosol pNR2B does not change (*n* = 3 per group; *P < 0.05, **P < 0.01, ***P < 0.001, TNBS *vs* sham, one-way ANOVA followed by LSD *post-hoc* test).

AMPAR and NMDAR expression are crucial for the induction and expression of pain-related LTP within the ACC, respectively [19]. Membrane and cytoplasmic proteins were isolated, and the distribution of N-cadherin, a specific marker of neural membrane, confirmed the successful separation of membrane and cytoplasmic proteins (Fig. 5A). As the key subunit of AMPARs, GluR1 generates AMPAR trafficking and integration within synaptic membranes [38]. Overexpression of GluR1 contributes to both somatic and visceral-related chronic pain [20, 39]. We found that the abundance of membrane GluR1 was markedly increased after TNBS treatment compared to that of the sham group (Fig. 5B, D), with no striking change in that of cytoplasmic GluR1 (Fig. 5C and E). Furthermore, the amount of membrane pGluR1 also increased during the course of CP (Fig. 5B, F), while that of cytoplasmic pGluR1 remained unchanged (Fig. 5C, G). These data suggest that the enhanced postsynaptic transmission in the ACC of TNBS-induced CP rats can be attributed to the recruitment and modification of the GluR1 subunit.

NR2B, the predominant NMDAR subunit in the ACC, undergoes long-term plastic changes under the condition of sustained pain [40]. Biochemical analysis of NR2B in the ACC at different time points showed that its membrane protein expression was significantly upregulated (Fig. 5B, H), while cytoplasmic expression was robustly down-regulated in CP rats compared to the sham group (Fig. 5C, I). In addition, membrane the expression of pNR2B was significantly ramped up during the course of CP (Fig. 5B, J). Despite a tendency of decreased cytoplasmic expression of pNR2B in the CP group, no significant difference was detected (Fig. 5C, K). These results suggest that TNBS induces membrane trafficking and modifies phosphorylation of the NR2B subunit in the ACC.

### Relief of Hyperalgesia *via* Microinjection of CNQX or AP-5 into the ACC

**Fig. 6:**
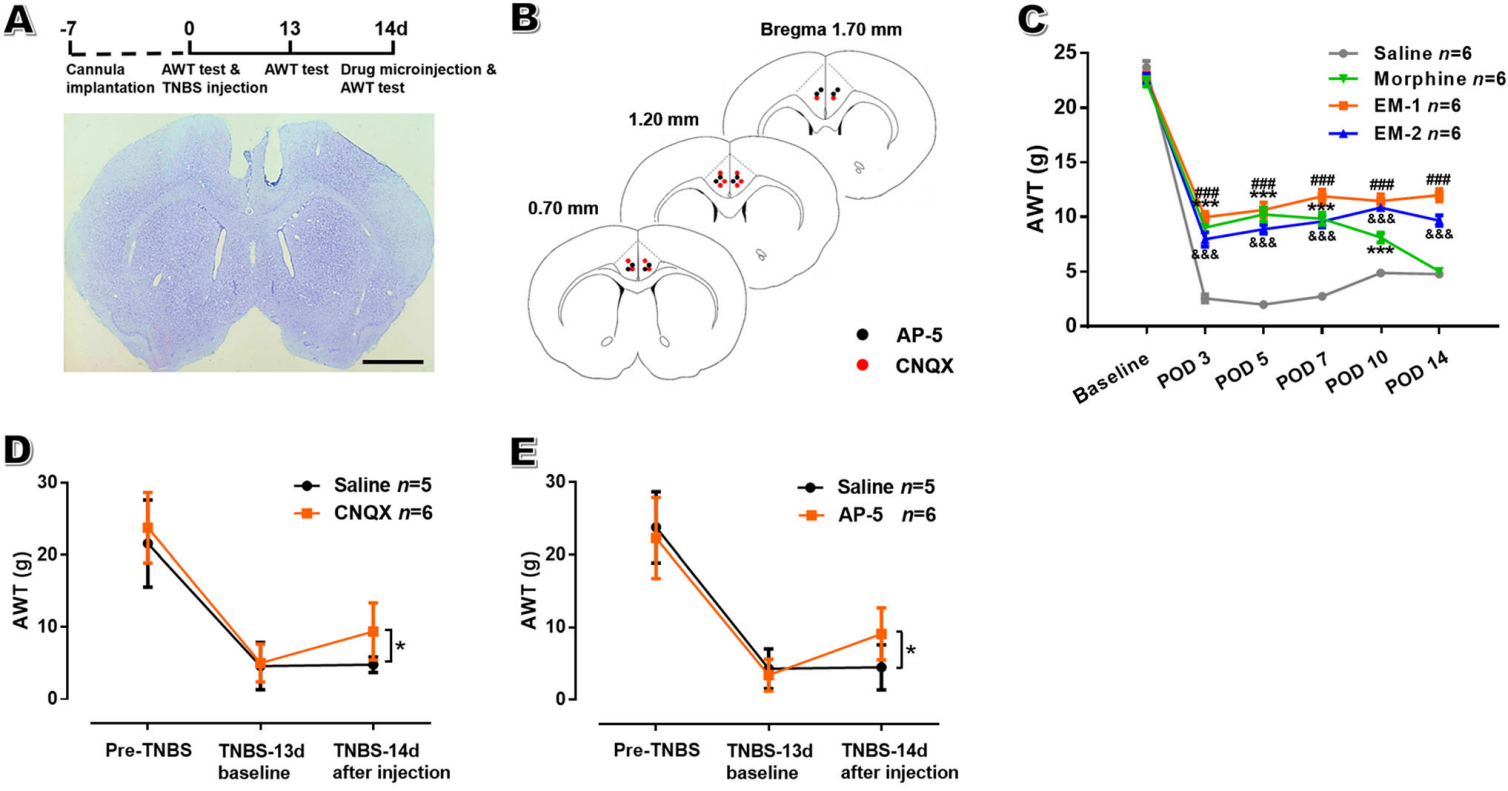
Bilateral microinjection of CNQX and AP-5 into the ACC alleviates abdominal hyperalgesia in CP rats. **A** Upper panel, schematic of behavioral experiment; lower panel, representative coronal section showing the sites of the cannulae in the ACC (scale bar, 2 mm). **B** Diagram showing the sites of the cannula tips for AP-5 (black dots) and CNQX (red dots) injection into the ACC. **C** Bilateral microinjections of morphine, EM-1, and EM-2 significantly increase the AWT in CP rats (*n* = 6 per group; ***P < 0.001, morphine *vs* saline; ^###^*P* < 0.001, EM-1 *vs* saline; ^&&&^*P* < 0.001, EM-2 *vs* saline, one-way repeated ANOVA followed by LSD *post-hoc* test). **D**, **E** Bilateral microinjections of CNQX (**D**) and AP-5 (**E**) on POD 14 significantly increase the AWT in CP rats but saline does not (*n* = 5 for saline-treated group and 6 for CNQX and AP-5-treated groups; *P < 0.05, TNBS *vs* sham unpaired *t*-test).

To determine the role of enhanced excitatory transmission in the ACC in the development of painful CP, the effects of local administration of CNQX and AP-5 to block ACC glutamatergic transmission on animal behaviors were measured by behavioral tests. Meanwhile, opioid receptor agonists that are known to elicit analgesia under chronic pain [41, 42] were microinjected into the ACC as a positive control. The experimental paradigm is illustrated in Fig. 6A, B. As shown in Fig. 6C, consecutive bilateral microinjection of EM-1 and EM-2 into the ACC had significant, stable analgesic effects in CP rats from POD 3 to POD 14 (AWT: 4.78 ± 0.32 g with saline *versus* 11.98 ± 0.57 g with EM-1 and 9.65 ± 0.49 g with EM-2, *P* < 0.001 for both, *n* = 6 per group). However, with morphine injection, there was a trend toward decreased analgesic effects from POD 3 to POD 10, indicating morphine tolerance. On POD 14, no significant difference was seen in the AWT between CP rats with morphine injection and saline injection.

Further behavioral data showed that bilateral microinjection of either CNQX (AWT: 4.80 ± 1.09 g, *n* = 5 with saline *versus* 9.40 ± 3.97 g, *n* = 6 with CNQX,* P* < 0.05; Fig. 6D) or AP-5 (AWT: 4.48 ± 3.11 g, *n* = 5 with saline *versus* 9.10 ± 3.60 g, *n* = 6 with AP-5,* P* < 0.05; Fig. 6E) into the ACC on POD 14 partly reversed the abdominal hyperalgesia in CP rat; this was equal to the effect of EM-1 or EM-2 injection into the ACC on POD 14. In accordance with our biochemical data, these behavioral results suggest that enhanced excitatory glutamatergic transmission in the ACC by postsynaptic recruitment of AMPARs and NMDARs contributes to abdominal hyperalgesia in CP rats.

### Relief of Hyperalgesia and Anxiety *via* Inhibiting Excitatory ACC Neurons

**Fig. 7:**
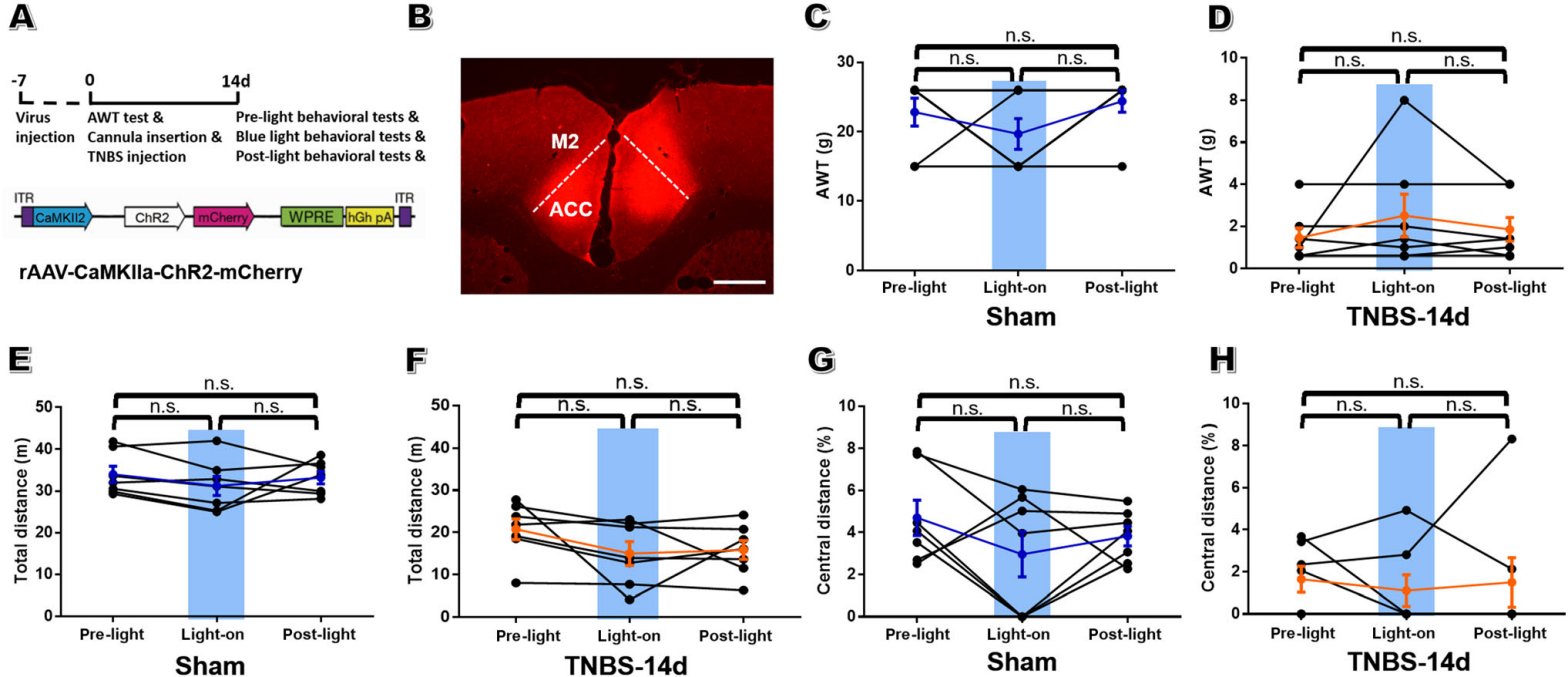
Optogenetic activation of bilateral ACC pyramidal neurons has no effect on AWT and anxiety-like behavior in sham and CP rats. **A** Upper panel, schematic of the behavioral experiment; lower panel, the rAAV-CaMKIIa-ChR2-mCherry construct. **B** Representative coronal section showing virus injection sites in the ACC (scale bar, 1 mm). **C**–**H** Activating bilateral pyramidal neurons in the ACC has no effect on the AWT (**C**, **D**), total distance (**E**, **F**), and the distance traveled in the center of the open field (**G**, **H**) in sham and CP rats (*n* = 7 for both groups; n.s., no significant difference, TNBS *vs* sham, one-way repeated ANOVA followed by LSD *post-hoc* test. ACC, anterior cingulate cortex; M2, secondary motor cortex.

**Fig. 8:**
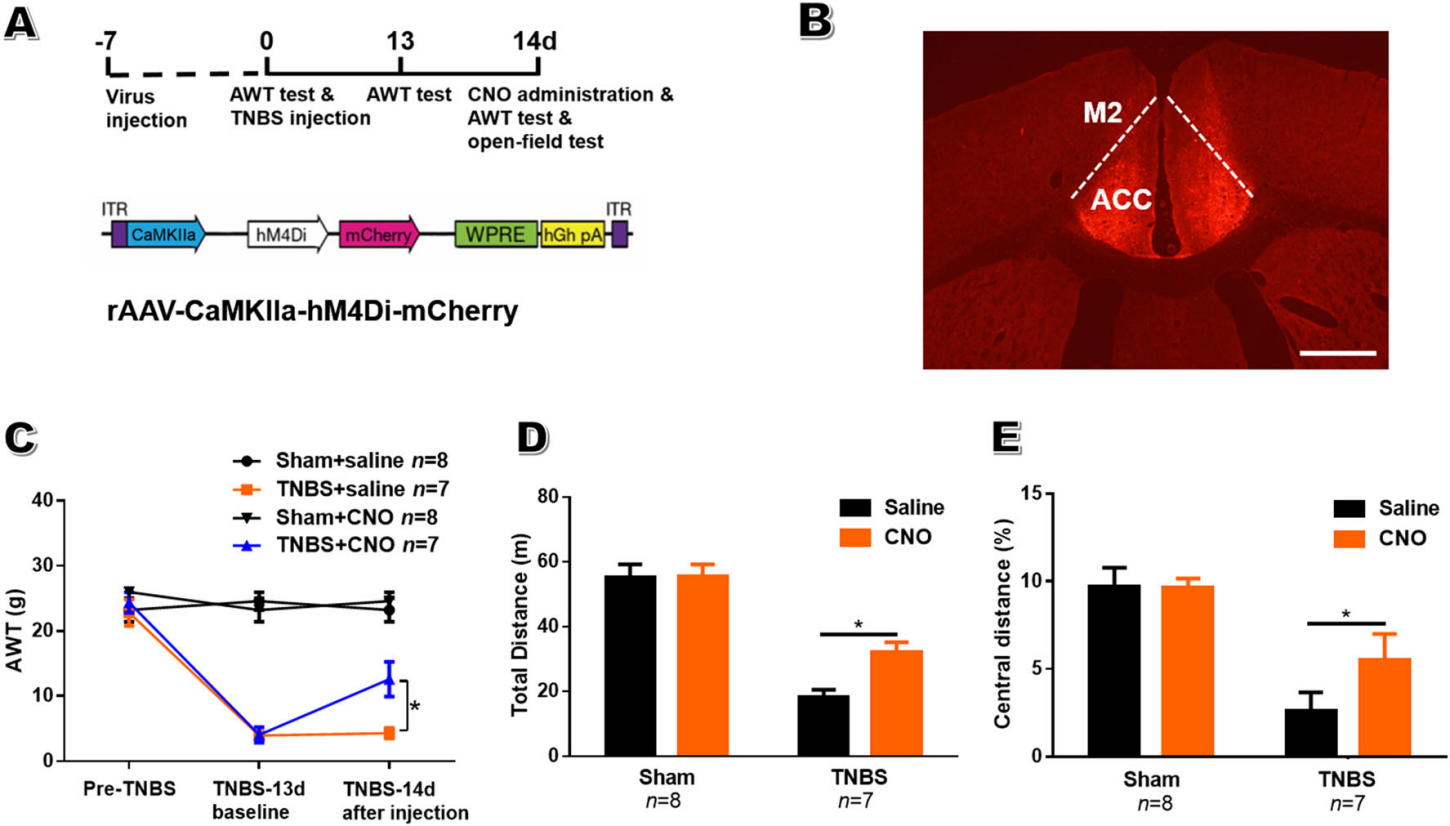
Chemogenetic inhibition of bilateral ACC pyramidal neurons alleviates abdominal hyperalgesia and anxiety-like behavior in CP rats. **A** Upper panel, schematic of the behavioral experiment; lower panel, rAAV-CaMKIIa-hM4Di-mCherry construct. **B** Representative coronal section showing the injection sites in the ACC (scale bar, 1 mm). **C** Inhibiting bilateral pyramidal neurons in the ACC *via* intraperitoneal CNO significantly increases the AWT in CP rats but not sham rats. **D**, **E** Inhibiting ACC neurons significantly reduces the total distance (**D**) and the distance traveled in the center (**E**) of the open field in CP rats but not sham rats (*n* = 8 for sham and 7 for CP rats; *P < 0.05, TNBS *vs* sham, unpaired *t*-test). ACC, anterior cingulate cortex; M2, secondary motor cortex.

Previous optogenetic studies have shown that excitatory pyramidal neurons are the primary components of the ACC to facilitate pain [43, 44], whereas interneurons play a diametrically opposite role [43]. Our morphological data have emphasized the role of ACC pyramidal neurons under the condition of painful CP. Thus, we propose that excitatory neurons mediate the pain-facilitatory role of the ACC in CP rats. In order to elucidate the role of pyramidal neurons in pancreatitis-related hyperalgesia and anxiety, we specifically modulated ACC pyramidal neurons bidirectionally *via* optogenetics and chemogenetics in sham and CP rats. The experimental protocols are shown in Figs 7A and 8A. ChR2-mCherry expression was validated by *post hoc* fluorescence microscopy (Fig. 7B). Behavioral results showed that the activation of pyramidal cells changed neither AWT (Fig. 7C–D) nor performance in the open field (Fig. 7E–H) in both sham and CP rats. hM4D(Gi)-mCherry expression was confirmed by *post hoc* fluorescence microscopy (Fig. 8B). On POD 14, CNO treatment alleviated the pancreatic hyperalgesia (AWT: 4.29 ± 0.81 g with TNBS+saline *versus* 12.57 ± 2.67 g with TNBS+CNO; *n* = 7 per group; *P* < 0.05; Fig. 8C) and decreased the anxiety-like behavior of TNBS-treated rats (total distance: 18.33 ± 2.17 m with TNBS+saline *versus* 32.16 ± 3.03 m with TNBS+CNO; % central distance: 2.58 ± 1.07 with TNBS+saline *versus* 5.49 ± 1.51 with TNBS+CNO; *n* = 7 per group; *P* < 0.05; Fig. 8D, E), but not in sham rats (Fig. 8C–E).

## Discussion

In the current study, we found that the ACC received direct projections from the NTS, a key relay station for primary visceral afferents, and this neural pathway was activated by painful CP. Specifically, morphological and biochemical results showed an increase in presynaptic glutamate release and postsynaptic glutamate receptor expression and phosphorylation in the ACC of CP rats, suggesting the existence of central sensitization under the condition of painful CP. These plastic changes contributed to abdominal hyperalgesia, since inhibiting excitatory transmission in the ACC induced significant analgesic effects comparable to local administration of opioids in CP rats. Finally, we further revealed that ACC pyramidal neurons mediated the behavioral representation of painful CP since chemogenetic inhibition of these neurons alleviated both hyperalgesia and pain-related anxiety in CP rats.

### TNBS-Induced Anxiety in CP Rats

In this study, chronic a pancreatitis model was established by intraductal administration of TNBS in rats. The mechanism by which this leads to chronic pancreatitis has not been fully clarified. It is generally accepted that as a hapten, TNBS reacts with lysine residues on the epithelium and then stimulates immunological responses against the tissue. TNBS may also have a direct toxic action by the formation of extremely reactive compounds with pro-inflammatory and cytotoxic properties, including superoxide and hydrogen peroxide radicals [45]. In this study, we found that TNBS-induced pathophysiological changes mimicked those seen in patients with chronic pancreatitis [46], providing a reliable painful CP animal model with the advantages of a high success rate, stability, and reproducibility along with less mortality.

As a common comorbidity, affective disorders under the condition of chronic pain severely impair patients’ quality of life and exacerbate the sensory abnormalities of chronic pain [47]. As expected, a high incidence of anxiety and depression is well-documented in patients with chronic pancreatitis [48]. Unfortunately, much less focus has been directed toward the relief of the affective dimension of pancreatitis-related pain in both clinical and preclinical studies. In this study, we found that CP rats exhibited hypolocomotion and decreased exploratory behavior both in the open field and elevated plus maze tests, consistent with prior behavioral observations in VH rats induced by irritable bowel syndrome and chronic pancreatitis [31, 34].

While decreased exploratory behavior is assumed to reflect anxiety in animal behavior experiments, immobility may be caused by many factors, including emotional disorders, motor disability, and decreased motor desire [33]. Here, the following lines of evidence suggest that negative emotions may be the sole reason for immobility in CP rats. First, it is recognized that chronic pain interferes with motor function [47]. Nevertheless, we failed to discriminate any alterations in motor performance between CP rats and sham rats in the rotarod test, thus excluding the possibility of motor deficits. Second, morphine treatment on POD 14 alleviated the abdominal hyperalgesia but failed to elevate motor performance in the open field teat (unpublished data), indicating that painful CP may not reduce motor desire in rats. Third, although clinical observations indicate that patients with CP exhibit somatic hypersensitivity in remote areas [14], we failed to observe somatic hypersensitivity of the hindpaw in CP rats, which differed from prior findings reported in CP mice [49]. Finally, the depression-like behavior in the forced swimming test further supports the presence of emotional disorders induced by CP. In light of these findings, hypolocomotion may be closely related to affective disorders under the condition of painful CP, and thus was used to measure the emotional aspect of pancreatic pain in this study. It is worth noting that decreased locomotion is seldom seen in neuropathic pain [47]. One possible explanation is that visceral pain is usually more unbearable than somatic pain and causes more severe emotional and autonomic disorders, which then lead to hypolocomotion.

### Involvement of the Direct NTS–ACC Pathway in Painful CP

The NTS is the primary target for afferent vagal fibers which convey noxious and non-noxious visceral sensory information to the forebrain *via* the parabrachial nucleus (PBN) [8, 9, 50]. The role of the NTS in the transmission or regulation of visceral or autonomic information has been extensively explored [51–54]. Ascending projections from the NTS have been studied in rodents with the aid of tract tracing techniques. Besides numerous brainstem territories including the PBN, the projections of the NTS have also been traced to a series of forebrain structures: the bed nucleus of the stria terminalis, the paraventricular, dorsomedial, and arcuate nuclei of the hypothalamus, the central amygdaloid nucleus, and the periventricular nucleus of the thalamus [55, 56]. Here, we demonstrated a direct NTS–ACC pathway in rats. Despite scarce projections, this connection provides evidence that the cortical pain center could be directly activated by visceral afferents processed from the NTS. Further observations indicated that the projections from the NTS were in close contact with both pyramidal and GABAergic ACC neurons, the former being more frequently seen. Another interesting phenomenon was that ACC pyramidal neurons were more likely to be activated by painful CP than GABAergic neurons. This morphological evidence suggests that ACC glutamatergic neurons play a crucial role in the transmission or modulation of painful CP, which was verified by subsequent functional studies. However, the role of GABAergic ACC neurons in the modulation of painful CP cannot be completely excluded, which is a limitation of this study. Further functional experiments are urgently needed to answer this question, which is an important research focus in our lab.

Apart from direct projections, there may be more complex indirect projections from NTS to ACC. As a key relay center for visceral afferents from the NTS, the PBN sends projections to extensive thalamic areas, including the intralaminar (centromedian, centrolateral) and the midline (paraventricular, reuniens) thalamic nuclei [57], which have been reported to send efferent connections to the ACC [58]. Thus, there may be an indirect NTS–PBN–thalamus–ACC pathway that is related to the transmission of visceral pain to the ACC. In addition, the NTS sends direct projections to the LC [55, 56], which provides direct norepinephrinergic efferents to the ACC [58]. Thus, the NTS–LC–ACC pathway may be involved in the modulation of visceral pain processing in the ACC. These potential indirect pathways warrant further investigation.

### Presynaptic and Postsynaptic Amplification of Painful CP in the ACC

LTP of glutamatergic transmission in the ACC is a key cellular mechanism for pathological pain [19]. Activity-dependent Ca^2+^ flux through NMDAR activation, induced by excessive presynaptic glutamate release, is thought to initiate downstream signaling events during LTP induction. Adenylate cyclase 1 (AC1) is one essential Ca^2+^-stimulated enzyme that converts ATP to cAMP and then activates downstream signaling molecules, such as protein kinase A (PKA) and cAMP-response element-binding protein (CREB). Evidence from neuropathic pain and VH models indicates that primary injuries promote GluR1 phosphorylation and trafficking in the ACC *via* the AC1–cAMP–PKA pathway, leading to NMDAR-mediated AMPAR potentiation [20, 59]. Meanwhile, activation of the AC1–cAMP–CREB pathway may facilitate NR2B expression and phosphorylation, forming a positive feedback to enhance NMDAR function and contribute to sustained pain [40]. Apart from AC1, calcium/calmodulin-dependent protein kinase II (CaMKII) is another crucial molecule for synaptic plasticity in the ACC. Phosphorylated CaMKII binds to and stabilizes postsynaptic NR2B, thus mediating visceral pain in VH rats [60].

In the present study, increased expression of VGluT1, as well as increased membrane trafficking and phosphorylation of GluR1 and NR2B subunits within the ACC were found in CP rats. We propose that CP-induced excessive glutamate release may activate similar signaling pathways *via* NMDARs in the ACC, leading to the recruitment of glutamate receptors and visceral hypersensitivity. These neuroplasticity-related changes within the ACC were positively associated with behavioral hyperalgesia under CP conditions, since suppression of glutamatergic transmission by AMPAR/NMDAR antagonists led to pain palliation, in accordance with previous studies performed under the conditions of neuropathic and gastrointestinal pain [20, 59]. We also tested the analgesic effects of local administration of opioids into the ACC in CP rats as a positive control for evaluating the analgesic effects of AMPAR/NMDAR antagonists and subsequent optogenetic/chemogenetic manipulations. Activating mu-opioid receptors (MORs) *via* morphine in the ACC has been shown to exert potent analgesic effects in various chronic pain states in rodents [41, 42, 61]. It has been proposed that MOR activation inhibits excitatory glutamatergic transmission in the ACC *via* the suppression of presynaptic glutamate release and the deactivation of pyramidal neurons [62, 63]. Here, we found that both exogenous (morphine) and endogenous (EM-1 and EM-2) MOR agonists had robust analgesic effects in CP rats, and these effects were almost equal to those elicited by AMPAR/NMDAR antagonists in the ACC. Taken together, the ACC plays a pain-facilitating role in pancreatitis-related pain. The limitation of our study is that other glutamate subunits that may be involved in painful CP have not been detected, and corresponding signaling mechanisms remain to be investigated.

### Neuromodulation Techniques Targeting the ACC as a Viable Therapy for Painful CP

In the present study, optogenetic modulation of ACC pyramidal cells failed to alter the AWT in naïve rats, which was inconsistent with Kang’s study which suggested that selectively activating excitatory ACC cells induces changes in the hindpaw mechanical threshold in naïve mice [43]. This discrepancy may be due to the limitation of our pain evaluation methods. Since VFF probing is a measure of referred abdominal mechanical hypersensitivity when pancreatic inflammation invades the peritoneum, it may not be an efficacious index of internal visceral sensation under normal conditions. Thus, recording defensive behaviors induced by pancreas stimulation *via* intra-abdominal electrodes [29] may be a better choice in studies to evaluate the sensory aspect of pancreatitis-related pain. In accordance with Kang’s study [43], we found that optogenetic activation of ACC pyramidal cells did not elicit anxiety-like behavior in normal rats, which differs from the results of Barthas [44]. Differences in the optogenetic virus, optogenetic stimulation protocol, and behavioral evaluation method may account for these conflicting results.

In CP, optogenetic activation of ACC pyramidal neurons did not aggravate the abdominal hyperalgesia or anxiety, suggesting a ceiling effects of the hyperalgesia induced by pancreatitis pain. In other words, the activity of excitatory ACC neurons reached a maximum during intense pancreatitis stimuli, so further enhancing their activity failed to elicit more pain sensation. This was supported by the fact that specifically inhibiting this neuron type had both anxiolytic and analgesic effects. Considering these, we conclude that glutamatergic ACC neurons mediate the abdominal hyperalgesia as well as the anxiety induced by chronic pancreatitis, and they are expected to be a promising target in the clinical treatment of painful CP. Another limitation in this study is that different methodologies were used to modulate ACC pyramidal neurons (e.g., using optogenetics for activation but chemogenetics for inhibition). These methods are not in strict contrast since the mechanisms of neuron responses are different [64]. Using one method to modulate neuronal activity bidirectionally (e.g., hM3Dq/hM4Di or ChR2/NpHR) may be better.

Historically, cingulectomy was introduced to treat intractable pain in clinical practice. Nevertheless, it is not currently recommended as a treatment due to its short-term effects and neuropsychiatric risks [18]. Instead, the advent of deep brain stimulation (DBS) provides a promising alternative for modifying dysfunctional pain-matrix activity in the treatment of refractory pain disorders, surpassing lesion surgery due to its adjustability and reversibility [65]. The ACC is a newly-identified stimulation spot for the treatment of drug-resistant depression [66] and chronic pain [67]. Bilateral ACC DBS is known to relieve various types of neuropathic pain and restore quality of life; it outperforms periaqueductal gray or thalamus stimulation by targeting the affective component of pain [66]. The predominant theory is that high-frequency stimulation generates depolarization blockade, leading to the functional inactivation of ACC neurons [59]. Interestingly, by controlling the neuronal activity of ACC, we successfully relieved pancreatitis-related pain and associated emotional disorders, which may pave the way for the application of ACC DBS in the treatment of refractory pancreatitis-related pain. However, neither pharmacologically nor chemogenetically blocking the activity of the ACC completely reversed the abdominal hyperalgesia in CP rats. This is understandable since the ACC occupies only one important node within the extensive cortical structures involved in pain and analgesia [68]. Other important areas, including sensory cortex and prefrontal cortex, are also involved in the cortical modulation of pain [69]. The role of these areas in the modulation of abdominal hyperalgesia and comorbid emotional disorders in CP rats remains to be investigated.

In summary, our results demonstrate that the ACC receives ascending projections from the NTS and that this pathway may be an essential portion of the ascending system involved in the transmission of abdominal hyperalgesia in CP rats. We also found that cortical sensitization plays a key role in abdominal hyperalgesia and pain-related negative emotions in rats with CP, and this was alleviated by inhibiting the excitability of pyramidal neurons in the ACC. These insights lay the foundation for prospective inquiries into detailed ACC-related circuit mechanisms in the hyperalgesia and anxiety induced by CP.

## Supplementary Information

Below is the link to the electronic supplementary material.

Supplementary file1 (PDF 614 KB)

## References

[1] Olesen SS, Juel J, Nielsen AK, Frøkjær JB, Wilder-Smith OH, Drewes AM (2014). Pain severity reduces life quality in chronic pancreatitis: Implications for design of future outcome trials. Pancreatology.

[2] Drewes AM, Krarup AL, Detlefsen S, Malmstrøm ML, Dimcevski G, Funch-Jensen P (2008). Pain in chronic pancreatitis: The role of neuropathic pain mechanisms. Gut.

[3] Fregni F, Pascual-Leone A, Freedman SD (2007). Pain in chronic pancreatitis: A salutogenic mechanism or a maladaptive brain response?. Pancreatology.

[4] Olesen SS, Krauss T, Demir IE, Wilder-Smith OH, Ceyhan GO, Pasricha PJ (2017). Towards a neurobiological understanding of pain in chronic pancreatitis: Mechanisms and implications for treatment. Pain Rep.

[5] Cervero F (1994). Sensory innervation of the viscera: Peripheral basis of visceral pain. Physiol Rev.

[6] Breit S, Kupferberg A, Rogler G, Hasler G (2018). Vagus nerve as modulator of the brain-gut axis in psychiatric and inflammatory disorders. Front Psychiatry.

[7] Pasricha PJ (2012). Unraveling the mystery of pain in chronic pancreatitis. Nat Rev Gastroenterol Hepatol.

[8] Berthoud HR, Neuhuber WL (2000). Functional and chemical anatomy of the afferent vagal system. Auton Neurosci.

[9] Hermes SM, Colbert JF, Aicher SA (2014). Differential content of vesicular glutamate transporters in subsets of vagal afferents projecting to the nucleus tractus solitarii in the rat. J Comp Neurol.

[10] Okada S, Katagiri A, Saito H, Lee J, Ohara K, Iinuma T (2019). Functional involvement of nucleus tractus solitarii neurons projecting to the parabrachial nucleus in trigeminal neuropathic pain. J Oral Sci.

[11] Zhuo M (2008). Cortical excitation and chronic pain. Trends Neurosci.

[12] de Vries M, Wilder-Smith OH, Jongsma ML, van den Broeke EN, Arns M, van Goor H (2013). Altered resting state EEG in chronic pancreatitis patients: Toward a marker for chronic pain. J Pain Res.

[13] Dimcevski G, Sami SA, Funch-Jensen P, Le Pera D, Valeriani M, Arendt-Nielsen L (2007). Pain in chronic pancreatitis: The role of reorganization in the central nervous system. Gastroenterology.

[14] Drewes AM, Gratkowski M, Sami SA, Dimcevski G, Funch-Jensen P, Arendt-Nielsen L (2008). Is the pain in chronic pancreatitis of neuropathic origin? Support from EEG studies during experimental pain. World J Gastroenterol.

[15] Frøkjær JB, Bouwense SA, Olesen SS, Lundager FH, Eskildsen SF, van Goor H (2012). Reduced cortical thickness of brain areas involved in pain processing in patients with chronic pancreatitis. Clin Gastroenterol Hepatol.

[16] Olesen SS, Frøkjær JB, Lelic D, Valeriani M, Drewes AM (2010). Pain-associated adaptive cortical reorganisation in chronic pancreatitis. Pancreatology.

[17] Lawal A, Kern M, Sanjeevi A, Hofmann C, Shaker R (2005). Cingulate cortex: A closer look at its gut-related functional topography. Am J Physiol Gastrointest Liver Physiol.

[18] Devinsky O, Morrell MJ, Vogt BA (1995). Contributions of anterior cingulate cortex to behaviour. Brain.

[19] Bliss TV, Collingridge GL, Kaang BK, Zhuo M (2016). Synaptic plasticity in the anterior cingulate cortex in acute and chronic pain. Nat Rev Neurosci.

[20] Liu SB, Zhang MM, Cheng LF, Shi J, Lu JS, Zhuo M (2015). Long-term upregulation of cortical glutamatergic AMPA receptors in a mouse model of chronic visceral pain. Mol Brain.

[21] Cao ZJ, Wu XY, Chen SL, Fan J, Zhang R, Owyang C (2008). Anterior cingulate cortex modulates visceral pain as measured by visceromotor responses in viscerally hypersensitive rats. Gastroenterology.

[22] Wu XY, Gao J, Yan J, Fan J, Owyang C, Li Y (2008). Role for NMDA receptors in visceral nociceptive transmission in the anterior cingulate cortex of viscerally hypersensitive rats. Am J Physiol Gastrointest Liver Physiol.

[23] Wang J, Zhang X, Cao B, Liu J, Li Y (2015). Facilitation of synaptic transmission in the anterior cingulate cortex in viscerally hypersensitive rats. Cereb Cortex.

[24] Zhou L, Huang JJ, Gao J, Zhang GP, Jiang JJ (2014). NMDA and AMPA receptors in the anterior cingulate cortex mediates visceral pain in visceral hypersensitivity rats. Cell Immunol.

[25] Fan J, Wu X, Cao Z, Chen S, Owyang C, Li Y (2009). Up-regulation of anterior cingulate cortex NR2B receptors contributes to visceral pain responses in rats. Gastroenterology.

[26] Yan N, Cao B, Xu JH, Hao C, Zhang X, Li Y (2012). Glutamatergic activation of anterior cingulate cortex mediates the affective component of visceral pain memory in rats. Neurobiol Learn Mem.

[27] Bai Y, Chen YB, Qiu XT, Chen YB, Ma LT, Li YQ (2019). Nucleus tractus solitarius mediates hyperalgesia induced by chronic pancreatitis in rats. World J Gastroenterol.

[28] Li J, Zhang MM, Tu K, Wang J, Feng B, Zhang ZN (2015). The excitatory synaptic transmission of the nucleus of solitary tract was potentiated by chronic myocardial infarction in rats. PLoS One.

[29] Winston JH, He ZJ, Shenoy M, Xiao SY, Pasricha PJ (2005). Molecular and behavioral changes in nociception in a novel rat model of chronic pancreatitis for the study of pain. Pain.

[30] Wan FP, Bai Y, Kou ZZ, Zhang T, Li H, Wang YY (2016). Endomorphin-2 inhibition of substance P signaling within *Lamina* I of the spinal cord is impaired in diabetic neuropathic pain rats. Front Mol Neurosci.

[31] Zhang MM, Liu SB, Chen T, Koga K, Zhang T, Li YQ (2014). Effects of NB001 and gabapentin on irritable bowel syndrome-induced behavioral anxiety and spontaneous pain. Mol Brain.

[32] Wang L, Wang J, Yang L, Zhou SM, Guan SY, Yang LK (2017). Effect of Praeruptorin C on 3-nitropropionic acid induced Huntington’s disease-like symptoms in mice. Biomedecine Pharmacother.

[33] Mogil JS, Crager SE (2004). What should we be measuring in behavioral studies of chronic pain in animals?. Pain.

[34] Bai Y, Ma LT, Chen YB, Ren D, Chen YB, Li YQ (2019). Anterior insular cortex mediates hyperalgesia induced by chronic pancreatitis in rats. Mol Brain.

[35] Coggeshall RE (2005). Fos, nociception and the dorsal horn. Prog Neurobiol.

[36] Wojcik SM, Rhee JS, Herzog E, Sigler A, Jahn R, Takamori S (2004). An essential role for vesicular glutamate transporter 1 (VGLUT1) in postnatal development and control of quantal size. Proc Natl Acad Sci USA.

[37] Morel L, Higashimori H, Tolman M, Yang YJ (2014). VGluT1+ neuronal glutamatergic signaling regulates postnatal developmental maturation of cortical protoplasmic astroglia. J Neurosci.

[38] Zhang JL, Abdullah JM (2013). The role of GluA1 in central nervous system disorders. Rev Neurosci.

[39] Qiu S, Zhang M, Liu Y, Guo YY, Zhao H, Song Q (2014). GluA1 phosphorylation contributes to postsynaptic amplification of neuropathic pain in the insular cortex. J Neurosci.

[40] Qiu S, Li XY, Zhuo M (2011). Post-translational modification of NMDA receptor GluN2B subunit and its roles in chronic pain and memory. Semin Cell Dev Biol.

[41] Wang LL, Hou KS, Wang HB, Fu FH, Yu LC (2020). Role of mu-opioid receptor in nociceptive modulation in anterior cingulate cortex of rats. Mol Pain.

[42] Gomtsian L, Bannister K, Eyde N, Robles D, Dickenson AH, Porreca F (2018). Morphine effects within the rodent anterior cingulate cortex and rostral ventromedial medulla reveal separable modulation of affective and sensory qualities of acute or chronic pain. Pain.

[43] Kang SJ, Kwak C, Lee J, Sim SE, Shim J, Choi T (2015). Bidirectional modulation of hyperalgesia via the specific control of excitatory and inhibitory neuronal activity in the ACC. Mol Brain.

[44] Barthas F, Sellmeijer J, Hugel S, Waltisperger E, Barrot M, Yalcin I (2015). The anterior cingulate cortex is a critical hub for pain-induced depression. Biol Psychiatry.

[45] Puig-Diví V, Molero X, Salas A, Guarner F, Guarner L, Malagelada JR (1996). Induction of chronic pancreatic disease by trinitrobenzene sulfonic acid infusion into rat pancreatic ducts. Pancreas.

[46] Adler G, Schmid RM (1997). Chronic pancreatitis: Still puzzling?. Gastroenterology.

[47] Liu MG, Chen J (2014). Preclinical research on pain comorbidity with affective disorders and cognitive deficits: Challenges and perspectives. Prog Neurobiol.

[48] Madan A, Borckardt JJ, Barth KS, Romagnuolo J, Morgan KA, Adams DB (2013). Interprofessional collaborative care reduces excess service utilization among individuals with chronic pancreatitis. J Healthc Qual.

[49] Cattaruzza F, Johnson C, Leggit A, Grady E, Schenk AK, Cevikbas F (2013). Transient receptor potential ankyrin 1 mediates chronic pancreatitis pain in mice. Am J Physiol Gastrointest Liver Physiol.

[50] Chamberlin NL, Mansour A, Watson SJ, Saper CB (1999). Localization of mu-opioid receptors on amygdaloid projection neurons in the parabrachial nucleus of the rat. Brain Res.

[51] Ramirez-Navarro A, Glazebrook PA, Kane-Sutton M, Padro C, Kline DD, Kunze DL (2011). Kv1.3 channels regulate synaptic transmission in the nucleus of solitary tract. J Neurophysiol.

[52] Freiria-Oliveira AH, Blanch GT, Li HW, Colombari E, Colombari DS, Sumners C (2013). Macrophage migration inhibitory factor in the nucleus of solitary tract decreases blood pressure in SHRs. Cardiovasc Res.

[53] Liu Y, Wen X, Liu SZ, Song DX, Wu D, Guan J (2015). KCa_1.1_-mediated frequency-dependent central and peripheral neuromodulation via Ah-type baroreceptor neurons located within nodose Ganglia and nucleus of solitary tract of female rats. Int J Cardiol.

[54] Liu Y, Zhao SY, Feng Y, Sun J, Lu XL, Yan QX (2020). Contribution of baroreflex afferent pathway to NPY-mediated regulation of blood pressure in rats. Neurosci Bull.

[55] Rinaman L (2010). Ascending projections from the caudal visceral nucleus of the solitary tract to brain regions involved in food intake and energy expenditure. Brain Res.

[56] Ricardo JA, Koh ET (1978). Anatomical evidence of direct projections from the nucleus of the solitary tract to the hypothalamus, amygdala, and other forebrain structures in the rat. Brain Res.

[57] Saper CB, Loewy AD (1980). Efferent connections of the parabrachial nucleus in the rat. Brain Res.

[58] Fillinger C, Yalcin I, Barrot M, Veinante P (2017). Afferents to anterior cingulate areas 24a and 24b and midcingulate areas 24a′ and 24b′ in the mouse. Brain Struct Funct.

[59] Xu H, Wu LJ, Wang HS, Zhang XH, Vadakkan KI, Kim SS (2008). Presynaptic and postsynaptic amplifications of neuropathic pain in the anterior cingulate cortex. J Neurosci.

[60] Li Y, Zhang X, Liu HY, Cao ZJ, Chen SL, Cao B (2012). Phosphorylated CaMKII post-synaptic binding to NR2B subunits in the anterior cingulate cortex mediates visceral pain in visceral hypersensitive rats. J Neurochem.

[61] LaGraize SC, Borzan J, Peng YB, Fuchs PN (2006). Selective regulation of pain affect following activation of the opioid anterior cingulate cortex system. Exp Neurol.

[62] Zheng WH (2010). Activation of mu opioid receptor inhibits the excitatory glutamatergic transmission in the anterior cingulate cortex of the rats with peripheral inflammation. Eur J Pharmacol.

[63] Chang WC, Lee CM, Shyu BC (2012). Temporal and spatial dynamics of thalamus-evoked activity in the anterior cingulate cortex. Neuroscience.

[64] Nectow AR, Nestler EJ (2020). Viral tools for neuroscience. Nat Rev Neurosci.

[65] Russo JF, Sheth SA (2015). Deep brain stimulation of the dorsal anterior cingulate cortex for the treatment of chronic neuropathic pain. Neurosurg Focus.

[66] Huebl J, Brücke C, Merkl A, Bajbouj M, Schneider GH, Kühn AA (2016). Processing of emotional stimuli is reflected by modulations of beta band activity in the subgenual anterior cingulate cortex in patients with treatment resistant depression. Soc Cogn Affect Neurosci.

[67] Xiao X, Ding M, Zhang YQ (2021). Role of the anterior cingulate cortex in translational pain research. Neurosci Bull.

[68] Treede RD, Kenshalo DR, Gracely RH, Jones AK (1999). The cortical representation of pain. Pain.

[69] Ohara PT, Vit JP, Jasmin L (2005). Cortical modulation of pain. Cell Mol Life Sci.

