# Supplementary material for: Anterior Cingulate Cortex Mediates Hyperalgesia and Anxiety Induced by Chronic Pancreatitis in Rats
Source: Neurosci Bull. 2021 Dec 15;38(4):342–58. doi: 10.1007/s12264-021-00800-x (PMC9068840; doi:10.1007/s12264-021-00800-x)
Supplement: Supplementary file 1 — Supplementary file1 (PDF 614 KB) [file 12264_2021_800_MOESM1_ESM.pdf]

Supplemental Figures and Figure Legends

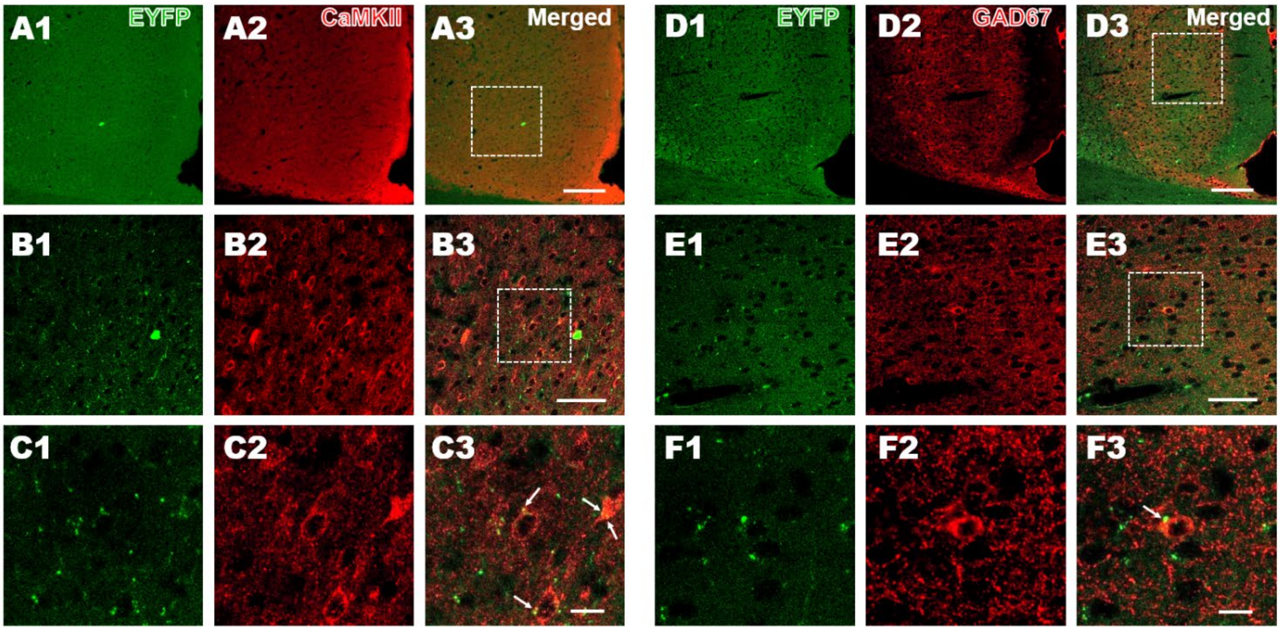

Fig. S1 Direct projections from the NTS are in close contact with CaMKII and GAD67-expressing neurons in the ACC. **A1, A2** EYFP-labeled terminals (**A1**, green) make close connections with CaMKII-expressing neural cell bodies (**A2**, red). **A3** Merged image from **A1** and **A2**. **B1–B3** Magnified images of the rectangles in **A1–A3. C1–C3** Magnified images of the rectangles in **B1–B3**. **D1, D2** EYFP-labeled terminals (**D1**, green) make close connections with a GAD67-expressing cell body (**D2**, red). **D3** Merged image from **D1** and **D2**. **E1–E3** Magnified images of the rectangles in **D1–D3**. **F1–F3** Magnified images of the rectangles in **E1–E3**. Arrows indicate the close connection sites between EYFP-labeled terminals and CaMKII-ir or GAD67-ir neurons [scale bars, 100 μm in **A1–A3** and **D1–D3**, 40 μm in **B1–B3** and **E1–E3**, and 10 μm in **C1–C3** and **F1–F3**.

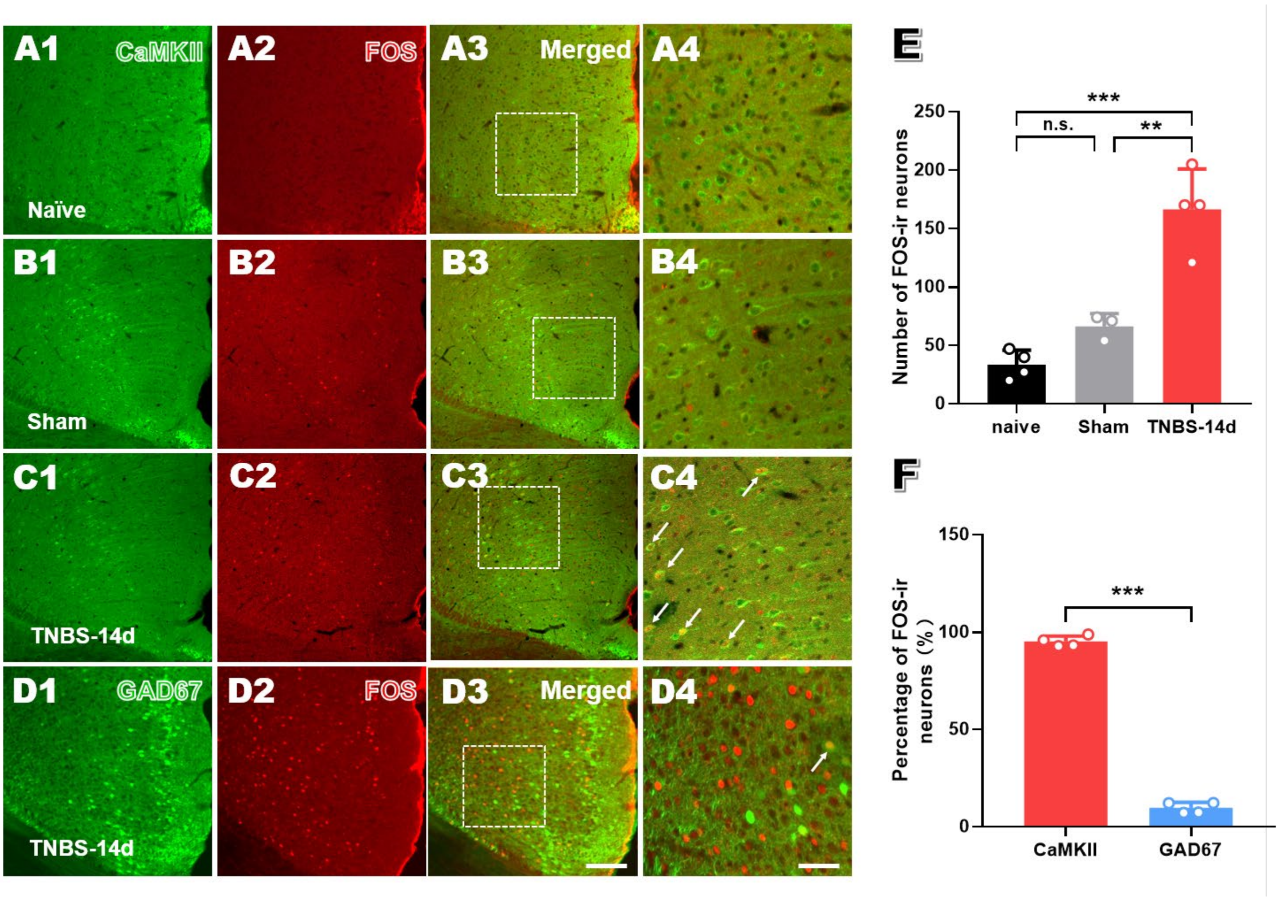

Fig. S2 A larger proportion of CaMKII-expressing neurons in the ACC express FOS than GAD-67-expressing neurons in CP rats. **A1–A3** CaMKII-expressing neurons (**A1**, green), FOS-expressing neurons (**A2**, red), and CaMKII/FOS double-labeled neurons (**A3**, yellow) in the ACC of naïve rats. **A4** Enlargement of the rectangle in **A3**. **B1–B3** CaMKII-expressing neurons (**B1**, green), FOS-expressing neurons (**B2**, red), and CaMKII/FOS double-labeled neurons (**B3**, yellow) in the ACC of sham rats. **B4** Enlargement of the rectangle in **B3**. **C1–C3** CaMKII-expressing neurons (**C1**, green), FOS-expressing neurons (**C2**, red), and CaMKII/FOS double-labeled neurons (**C3**, yellow) in the ACC of CP rats. **C4** Enlargement of the rectangle in **C3**. **D1–D3** GAD-67-expressing neurons (**D1**, green), FOS-expressing neurons (**D2**, red), and GAD-67/FOS double-labeled neurons (**D3**, yellow) in the ACC of CP rats. **D4** Enlargement of the rectangle in **D3**. Arrows indicate double-labeled neurons [scale bars, 100 μm in **A1–A3**, **B1–B3**, **C1–C3**, and **D1–D3**, and 60 μm in **A4**, **B4**, **C4**, and (**D4**). **E** Numbers of FOS-ir neurons in the ACC in rats from different groups. The number of FOS-ir neurons in the ACC is significantly higher in CP rats than in naïve and sham rats, while there is no difference in the numbers of FOS-ir neurons between naïve and sham rats. **F** Percentages of CaMKII/FOS double-labeled neurons and GAD67/FOS double-labeled neurons in the ACC of CP rats. A larger proportion of

CaMKII-expressing neurons in the ACC express FOS than GAD-67-expressing neurons in CP rats (*n* = 4 for all groups; $^{**}P<0.01$, $^{***}P<0.001$, n.s., no significant difference, one-way ANOVA followed by LSD *post-hoc* test in **E**, paired *t*-test in **F**).
